# A Bibliometric and Knowledge-Map Analysis of CAR-T Cells From 2009 to 2021

**DOI:** 10.3389/fimmu.2022.840956

**Published:** 2022-03-18

**Authors:** Lele Miao, Juan Zhang, Zhengchao Zhang, Song Wang, Futian Tang, Muzhou Teng, Yumin Li

**Affiliations:** ^1^Department of General Surgery, Second Hospital of Lanzhou University, Lanzhou, China; ^2^Key Laboratory of the Digestive System Tumors of Gansu Province, Lanzhou, China; ^3^Department of Hematology, Fifth Medical Center, Chinese People's Liberation Army (PLA) General Hospital, Beijing, China; ^4^Lanzhou University, Lanzhou, China

**Keywords:** CAR-T cell, Citespace, VOSviewer, bibliometric, knowledge-map, hotspots, topics

## Abstract

**Objectives:**

A bibliometric and knowledge-map analysis is used to explore hotspots’ evolution and development trends in the CAR-T cell field. By looking for research hotspots and new topics, we can provide new clues and ideas for researchers in this field.

**Methods:**

The articles and reviews regarding CAR-T cells were retrieved and obtained from the Web of Science Core Collection (WOSCC) on October 28th, 2021. CtieSpace [version 5.8.R3 (64-bit)] and VOSviewer (version 1.6.17) were used to conduct the bibliometric and knowledge-map analysis.

**Results:**

660 authors from 488 institutions in 104 countries/regions published 6,867 papers in 1,212 academic journals. The United States was absolutely in the leading position in this research field. The institution that contributed the most publications was the University of Pennsylvania. Carl H June published the most articles, while Shannon L Maude had the most co-citations. However, there was little cooperation between countries. After 2012, cooperation among various institutions was also small. The journals that published the most CAR-T cell-related papers were *Frontiers in immunology* and *Cancers*. Nevertheless, *Blood* and *The New England Journal of Medicine* were the most commonly co-cited journals. The most influential research hotspots were the research of CAR-T cells in hematological malignancies, the related research of cytokine release syndrome (CRS), CD19, and the anti-tumor activity and efficacy of CAR-T cells. The latest hotspots and topics included the study of CAR-T cells in solid tumors, universal CAR-T cells, CAR-NK cells, CD22, and anakinra (the IL-1 receptor antagonist). The research of CAR-T cells in solid tumors was a rapidly developing hot field. Emerging topics in this field mainly included the study of CAR-T cells in glioblastoma (related targets: IL13Rα2, EGFRvIII, and HER2), neuroblastoma (related target: GD2), sarcoma (related target: HER2), and pancreatic cancer (related target: mesothelin), especially glioblastoma.

**Conclusion:**

As an anti-tumor therapy with great potential and clinical application prospects, CAR-T cell therapy is still in a stage of rapid development. The related field of CAR-T cells will remain a research hotspot in the future.

## Introduction

CAR-T cell therapy has developed rapidly in recent years as promising adoptive immunotherapy. It is mainly used to research and treat malignant tumors, especially hematological malignant tumors, and has achieved stimulating clinical effects. In recent 20 years, this therapy has made significant progress in many aspects, mainly in the following aspects: a. CARs have been developed from the first generation to the fifth generation; b. The application of CAR-T cells has been gradually expanded the research and treatment of solid tumors. Compared with hematological malignancies, the biological characteristics of solid tumors are more complex so that CAR-T cells will face more obstacles and challenges in solid tumors (1); c. Improving the efficacy and/or safety of CAR-T cells, some special CARs have been developed based on traditional CARs, such as tandem CARs (2–5), syNotch CARs (6–8), inhibitory CARs (iCARs) (9), AND-NOT CARs (10), and ON/OFF-switch CARs (11–14). d. From single CAR-T cell therapy to combination therapy, for example, combined chemotherapy (15, 16), radiotherapy (17–19), or immune checkpoint inhibitors (20, 21).

As an anti-tumor therapy, the goal of CAR-T cell therapy is clinical transformation and clinical application. In recent years, there has been more and more basic research and clinical research related to CAR-T cells and more papers. Many scholars have reviewed this therapy from many aspects, including how to improve the efficacy and safety of CAR-T cells (22–24), the mechanism and management of related toxic reactions (25–28), the improvement and optimization of CAR structure (29, 30), the selection of targets (31, 32), the influence of TME on CAR-T cells (33, 34), and the research of CAR-T cells in hematological malignancies or solid tumors (35–38).

Bibliometrics is an interdisciplinary science that uses mathematical and statistical methods to analyze knowledge carriers, such as literature quantitatively. Through the comprehensive and objective analysis of most literature on a specific topic by bibliometrics, we can get some vital information. The information includes: a. the contribution of countries/regions, institutions, journals, and authors in this field; b. the collaboration between countries, institutions, or authors; c. the distribution of journals; d. the knowledge base (39–41). Therefore, bibliometrics can help researchers quickly understand a particular field, including research hotspots and evolving trends in this field, and avoid repeated research (42–44).

In this study, CiteSpace [version 5.8.R3 (64-bit)] and VOSviewer (version 1.6.17) were used to analyze the CAR-T cell-related literature and draw the scientific knowledge maps. This study aimed to explore the evolution and development trend of research hotspots in the CAR-T cell field from 2009 to 2021 and seek new hotspots and topics. It was hoped that this study would provide new clues and ideas for the subsequent study of CAR-T cells.

## Materials and Methods

### Data Collection

The data was retrieved and downloaded from WoSCC (Lanzhou University Purchase Edition) on October 28th, 2021. We set the search formula: TS= (CAR-T OR CAR T cell OR CAR-T cell OR CAR T-cell OR CAR-T-cell OR chimeric antigen receptor T cell OR chimeric antigen receptor-T cell OR chimeric antigen receptor T-cell OR chimeric antigen receptor-modified T-cell OR chimeric antigen receptor-transduced T-cell OR chimeric antigen receptor-redirected T cell OR chimeric antigen receptor redirecting T-cell OR chimeric antigen receptor engineered T cell OR chimeric antigen receptor-engineered T-cell). The retrieval time range was from 1980 to October 28th, 2021, and the language was limited to English. The article type was limited to article or review. 7,806 papers (no duplicate) were obtained, including 4,862 articles and 2,944 reviews. Perhaps due to a large amount of data, some functions of CtieSpace [version 5.8.R3 (64-bit)] ran very slowly, so we narrowed the search scope to reduce some literature. We searched in the same way as above, limiting the time from January 1st, 2009 to October 28th, 2021. A total of 6867 papers (no duplicate) were obtained, including 3,980 articles and 2,887 reviews. Except for “The Annual Growth Trend of Publication Outputs”, we used the first set of data (years:1980-2021) for analysis; for other studies, we adopted the second set of data (years: 2009-2021) to analyze. It should be mentioned that the second group of data is also significant, including 6867 papers (accounting for 88% of all papers). The retrieved papers were exported in the form of “Full Record and Cited References” and saved in “Plain Text”. In addition, these files were named “download_.txt”.

### Data Analysis

Microsoft Office Excel 2010 was used to manage data and analyze annual publications. Besides, we also used CtieSpace [version 5.8.R3 (64-bit)] and VOSviewer (version 1.6.17) to analyze these data and draw scientific knowledge maps visually.

CtieSpace is a JAVA-based citation visualization software developed by Chaomei Chen, which provides an experimental platform for researching new ideas and comparing existing methods (45). It is one of the most commonly used visual analysis software in bibliometrics. It can analyze the potential literature from multiple angles, observe the research hotspots and trends in a specific field, and visually present them. The knowledge-map can help researchers intuitively understand the research hotspots and evolution process and forecast the research and development trend of the field of interest (46).

VOSviewer is a free JAVA-based software for bibliometric mapping developed by Nees Jan van Eck and Ludo Waltman in 2009. It focuses more on the visualization of scientific knowledge (47). Moreover, VOSviewer has a powerful ability to handle large maps, which can display large bibliometric maps in an easily interpretable way (47).

## Results

### The Annual Growth Trend of Publication Outputs

**Figure 1:**
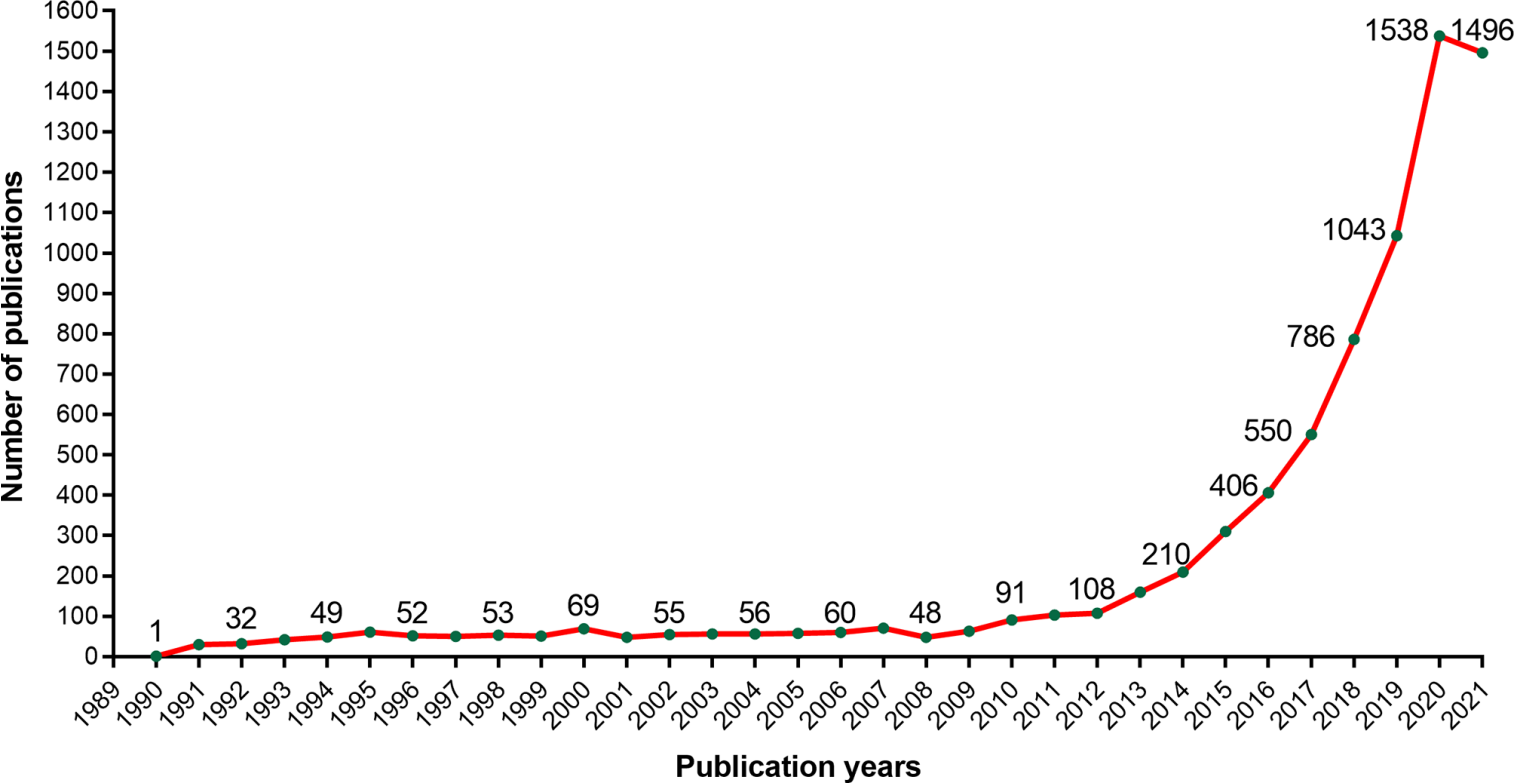
The trend of publication outputs about CAR-T cells.

We could know the development trend by counting the CAR-T cells published every year. Setting the retrieval time range from 1980 to October 28th, 2021, we got 7806 papers related to CAR-T cells from WoSCC **(Annexes 1)**. As shown in **Figure 1**, the publications about CAR-T cells are increasing year by year. From 1990 to 2009, the output of publications in this period was meagre, and From 2009 to 2012, the number of relevant papers showed a slow upward trend. From 2012 to 2020, the number of related papers increased rapidly, of which 1,538 papers were published in 2020. By October 28th, 1,496 relevant papers had been published in 2021.

Furthermore, we adopted the second set of data (**Annexes 2**) to analyze. The reasons we had been mentioned in “*Data Collection*” above.

### Countries/Regions and Institutions

A total of 488 institutions from 104 countries/regions co-authored 6,867 publications. As can be seen from **Table 1**, the country with the largest output of publications in the United States (n=3554, accounting for 51.8% of the total), followed by China (n=1253, 18.2%), Germany (n=703, 10.2%), England (n=405, 5.9%) and Italy (n=353, 5.14%). The number of publications from the United States far exceeded other countries. Among the top 10 countries, England, Italy, and France had higher centrality, 0.31, 0.54, and 0.4, respectively. It showed that these countries played a strong role as a bridge in the cooperation between countries. 90% of the top 10 countries with the most publications were developed countries. The institution that contributed the most publications was Univ Penn (n=419, 6.10%), followed by Mem Sloan Kettering Canc Ctr (n=299, 4.35%), Univ Texas MD Anderson Canc Ctr (n=263, 3.83%), NCI (n=213, 3.10%), and Univ Washington (n=208, 3.03%). It was worth noting that the top 10 institutions were all from the United States.

**Table 1:** The top 10 countries/regions and institutions involved in CAR-T cells.

Rank	country/region	Count	Centrality	Year	Institution	Count	Centrality	Year
1	USA	3554	0.1	2009	Univ Penn (USA)	419	0.1	2009
2	PEOPLES R CHINA.	1253	0.03	2009	Mem Sloan Kettering Canc Ctr (USA)	299	0.03	2009
3	GERMANY.	703	0.16	2009	Univ Texas MD Anderson Canc Ctr (USA)	263	0.13	2009
4	ENGLAND.	405	0.31	2009	NCI (USA)	213	0.03	2009
5	ITALY.	353	0.54	2009	Univ Washington (USA)	208	0.02	2009
6	FRANCE.	260	0.4	2009	Harvard Med Sch (USA)	194	0	2016
7	JAPAN.	210	0.1	2009	Baylor Coll Med (USA)	168	0.03	2009
8	CANADA.	199	0.1	2010	Fred Hutchinson Canc Res Ctr (USA)	165	0.23	2009
9	AUSTRALIA.	173	0.03	2009	Stanford Univ (USA)	120	0.03	2009
10	SPAIN.	167	0.1	2009	Mayo Clin (USA)	108	0	2010

**Figure 2:**
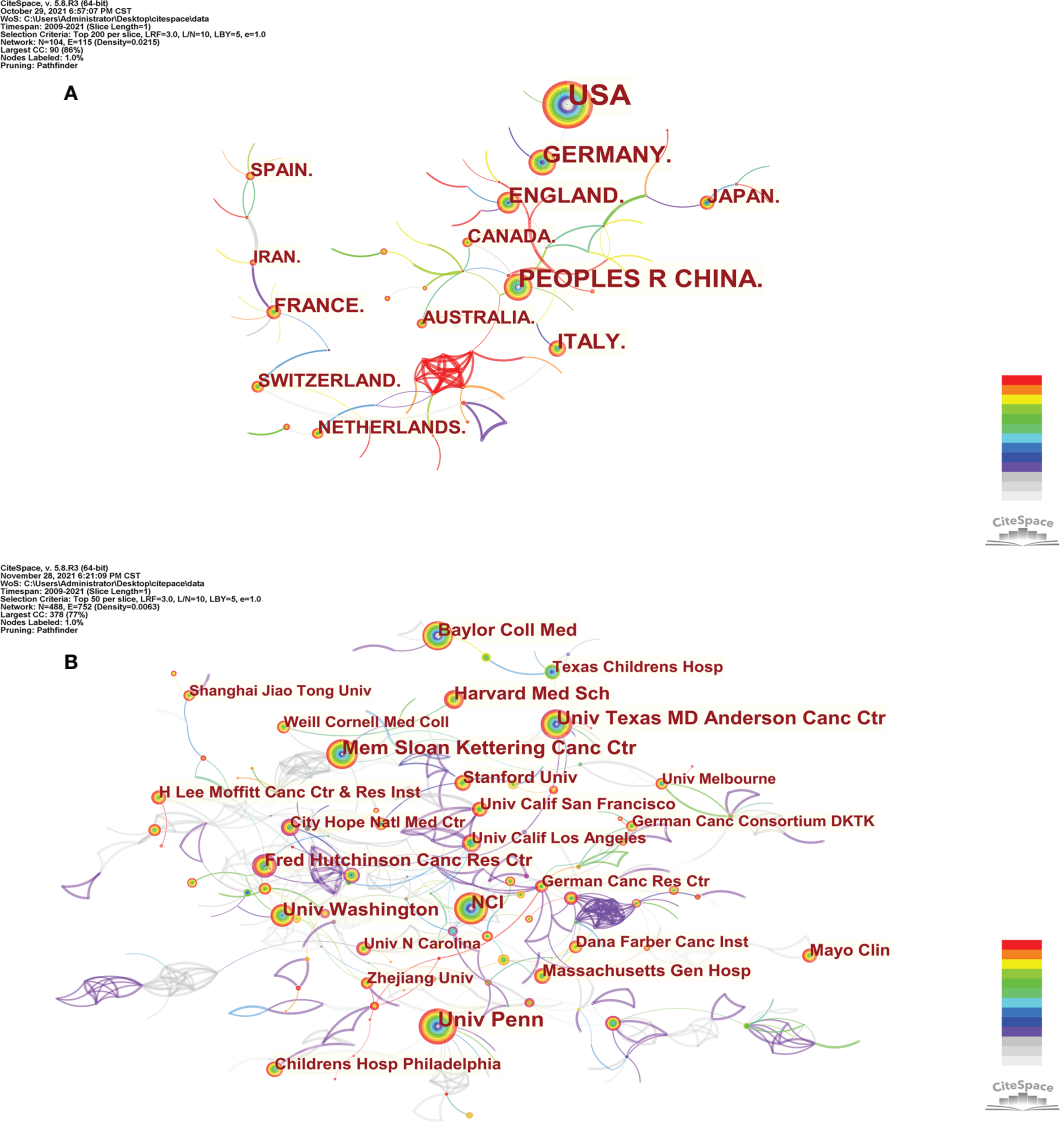
The co-occurrence map of countries/regions **(A)** and institutions **(B)** in CAR-T cell research.

As shown in **Figure 2A**, the connection between countries is sparse, indicating that there is little cooperation between countries. From **Figure 2B**, we can see that the purple and gray connections are the most, which indicates that the most intensive years of inter-agency cooperation are 2012 and before 2012, and there is little inter-agency cooperation after 2012.

### Journals and Co-Cited Journals

**Figure 3:**
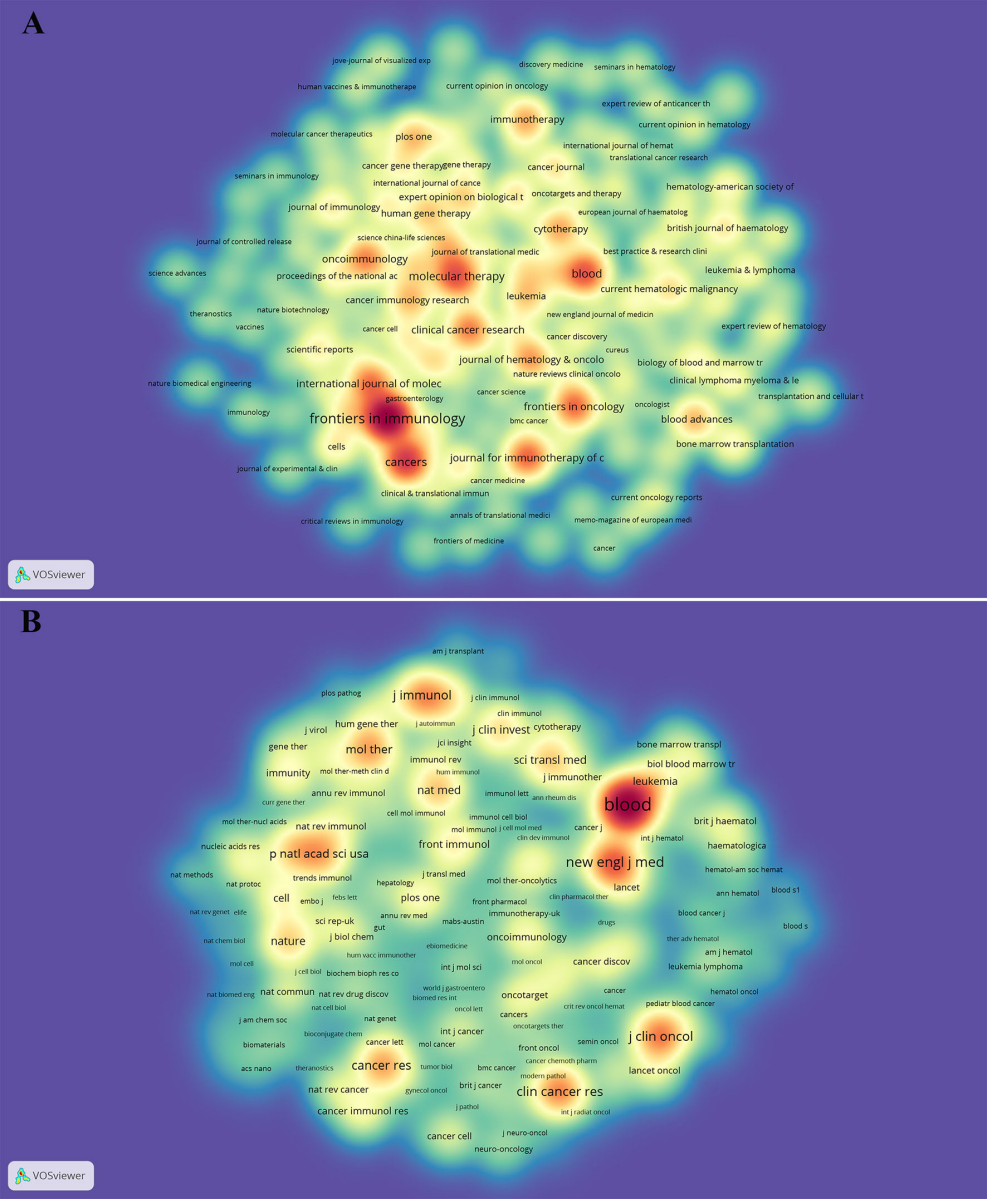
The density map of journals **(A)** and co-cited journals **(B)** in CAR-T cell research. **(A)** shows journals with several publications ≥10; **(B)** shows the journals with citations ≥200.

VOSviewer (version 1.6.17) and CtieSpace [version 5.8.R3 (64-bit)] were used to perform the co-citation and co-cited journal analysis, and finally found the journals with the most published papers and the journals with the most co-citations in this field. The results showed that 6867 papers were published in 1,212 academic journals. **Table 2** shows that the most published papers are *Frontiers in Immunology* (n=296), followed by *Cancers* (n=169), *Molecular Therapy* (n=137), *Blood* (n=134), and *Journal for Immunotherapy of Cancer* (n=118). Among the top 10 journals, eight had published more than 100 papers, and six were located in the Q1 Journal Citation Reports (JCR) region. The density map can well show the most published journals (**Figure 3A**). In addition, among these journals, the impact factor (IF) of *Blood* (IF=23.629) was highest.

**Table 2:** Top 10 journals and co-cited journals related to CAR-T cells.

Rank	Journal	Count	IF(2020)	JCR(2020)	Co-cited journal	Citation	IF(2020)	JCR(2020)
1	Frontiers in immunology	296	7.561	Q2	Blood	46825	23.629	Q1
2	Cancers	169	6.639	Q2	New england journal of medicine	20112	91.253	Q1
3	Molecular therapy	137	11.454	Q1	Journal of clinical oncology	16127	44.544	Q1
4	Blood	134	23.629	Q1	Clinical cancer research	15253	12.531	Q1
5	Journal for immunotherapy of cancer	118	13.751	Q1	Journal of Immunology	13652	5.422	Q1
6	Frontiers in oncology	115	6.244	Q2	Cancer research	12180	12.701	Q1
7	International journal of molecular sciences	107	5.924	Q3	Molecular therapy	11821	11.454	Q1
8	Clinical cancer research	102	12.531	Q1	Proceedings of the national academy of sciences of the united states of america	10602	11.205	Q1
9	Oncoimmunology	97	8.110	Q1	Nature medicine	9392	53.440	Q1
10	Journal of hematology & oncology	87	17.388	Q1	Science	8934	47.728	Q1

IF, Impact Factor; JCR, Journal citation reports.

As can be seen from **Table 2**, the most frequently cited journals are *Blood* (n=46825), followed by *New England Journal of Medicine* (n=20112), *Journal of Clinical Oncology* (n=16127), *Clinical Cancer Research* (n=15253), and *Journal of Immunology* (n=13562). Among the top 10 co-cited journals, 8 journals were cited more than 10,000 times, and the cited times of *Blood* far exceeded those of other journals. The density map can well show the most cited journals (**Figure 3B**). Among these journals, 9 journals had an impact factor greater than 11. Among them, the journals with the highest IF were the *New England Journal of Medicine* (IF=91.253), followed by *Nature Medicine* (IF=53.440), *Science* (IF=47.728), *Journal of Clinical Oncology* (IF=44.544), and *Blood* (IF=23.629).

**Figure 4:**
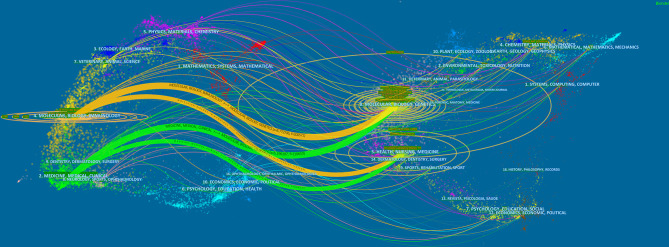
The dual-map overlay of journals on CAR-T cells. Image parameter: a: 2; Source Circle Size: 25; Target Circle Size: 3; Snap to centroids (< Radius): 0. The citing journals are located on the left, and the cited journals are located on the right. The color paths (two orange and two green reference paths) represent the cited relationship.

The dual-map overlay of journals can well show the distribution of journals and the relationship between journals and cited journals (the color path represents the cited relationship) (48). **Figure 4** identifies four main reference paths. It indicated that papers published in “Molecular, Biology, Genetics” journals and “Health, Nursing, Medicine” journals were often cited in papers published in “Molecular, Biology, Immunology” journals and “Medicine, medical, Clinical” journals.

### Authors and Co-Cited Authors

**Figure 5:**
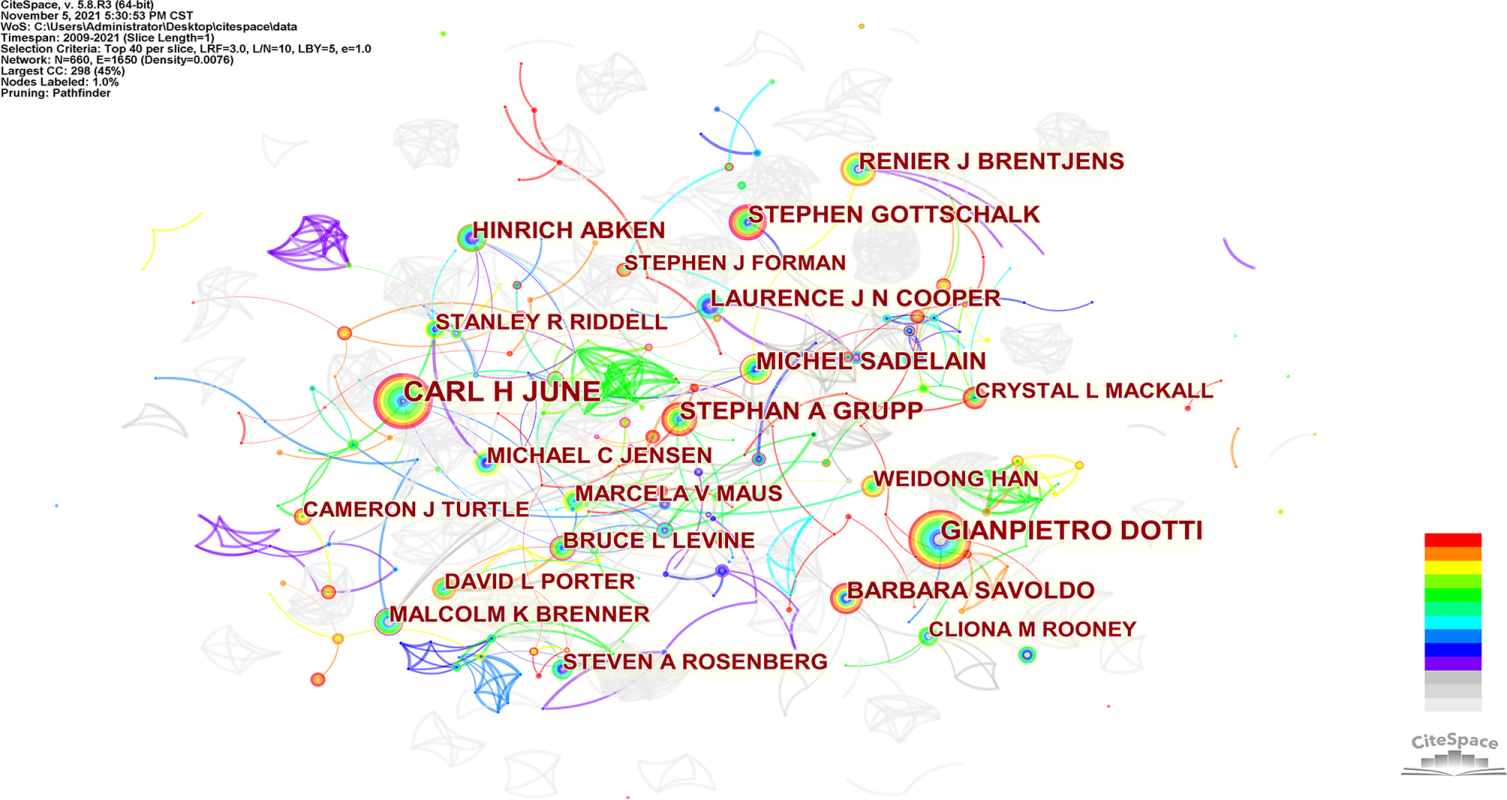
CiteSpace visualization map of authors involved in CAR-T cells.

660 authors co-authored 6,867 publications. As shown in **Table 3**, the most published papers are Car H June (n=133), followed by Gianpietro Dotti (n=97), Stephan A Grupp (n=68), Michel Sadelain (n=62), and Stephen Gottschalk (n=55). The centrality of the top 10 authors was not high. Only Car H June (0.14) and Michel Sadelain (0.14) were more significant than 0.10. From **Figure 5**, we can see a certain degree of cooperation between different authors. Each circle represents one author, and the lines between circles represent cooperation among authors; thicker lines mean closer cooperation, and different colors represent different years.

**Table 3:** The top 10 authors and co-cited authors of CAR-T cell research.

Rank	Author	Count	Centrality	Co-cited author	Citation	Centrality
1	CARL H JUNE	133	0.14	MAUDE SL	2446	0.55
2	GIANPIETRO DOTTI	97	0.01	KOCHENDERFER JN	1827	0.6
3	STEPHAN A GRUPP	68	0.03	LEE DW	1729	0.28
4	MICHEL SADELAIN	62	0.14	PORTER DL	1652	0
5	STEPHEN GOTTSCHALK	55	0.1	BRENTJENS RJ	1421	0.64
6	BARBARA SAVOLDO	54	0.07	NEELAPU SS	1369	0.1
7	RENIER J BRENTJENS	49	0.01	MORGAN RA	1359	0.3
8	HINRICH ABKEN	48	0.03	GRUPP SA	1312	0.62
9	LAURENCE J N COOPER	48	0	DAVILA ML	1189	0.04
10	MARCELA V MAUS	43	0.03	TURTLE CJ	1119	0.08

**Figure 6:**
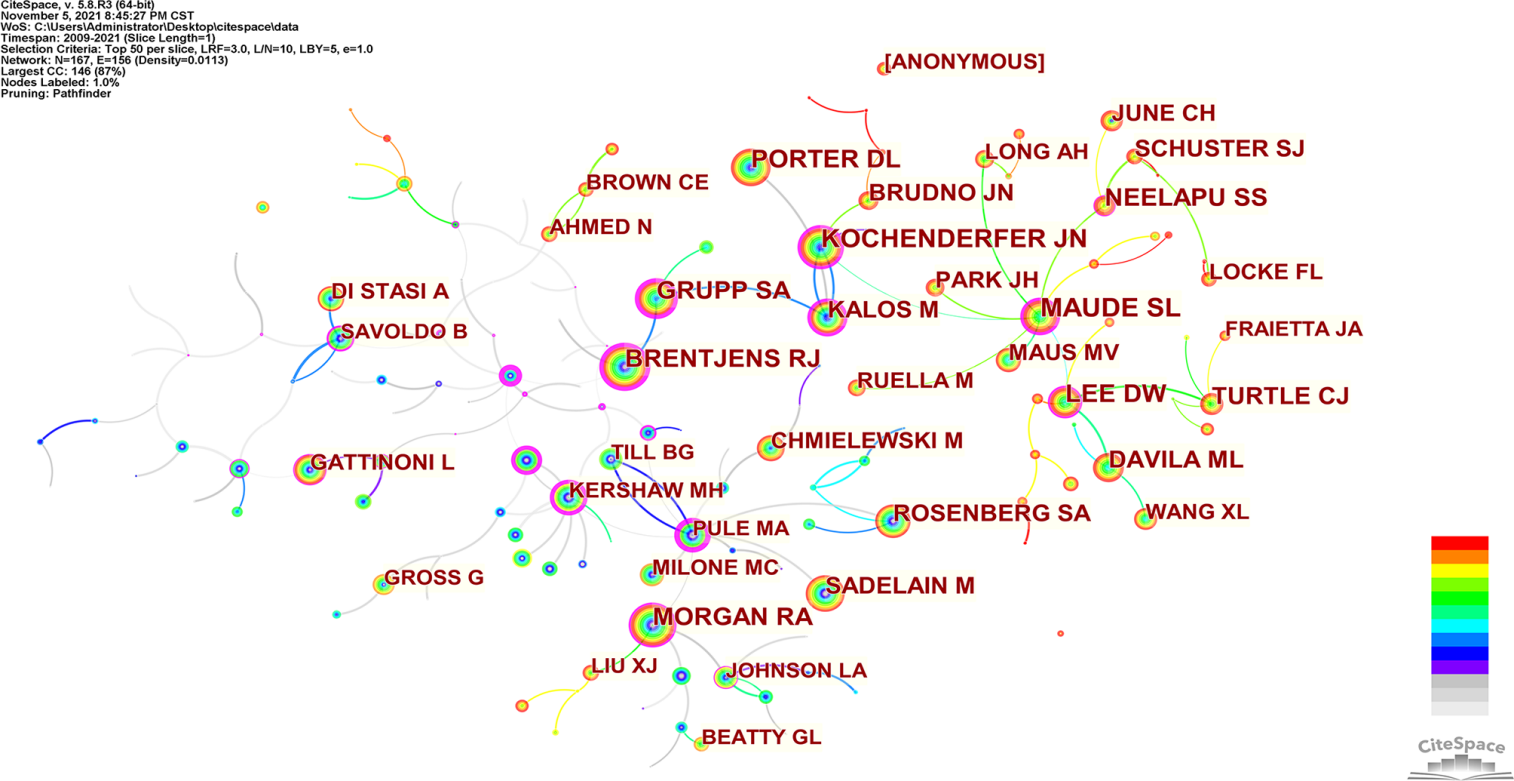
CiteSpace visualization map of co-cited authors involved in CAR-T cells.

Co-cited authors are two (or more) authors cited by one or more papers simultaneously. As shown in **Table 3**, the top 10 co-cited authors are cited more than 1000 times. The most frequently co-cited authors are Maude SL (n=2446), followed by Kochenderfer JN (n=1827), Lee DW (n=1729), Porter DL (n=1652) and Brentjens RJ (n=1421). Among the top 10 authors, there were 6 whose centrality exceeded 0.10, of which Brentjens RJ (0.64) was the highest. These co-cited authors with high centrality show annual purple rings in **Figure 6**, indicating that they have played an important role as a bridge.

### Keyword Co-Occurrence, Clusters, and Evolution

**Figure 7:**
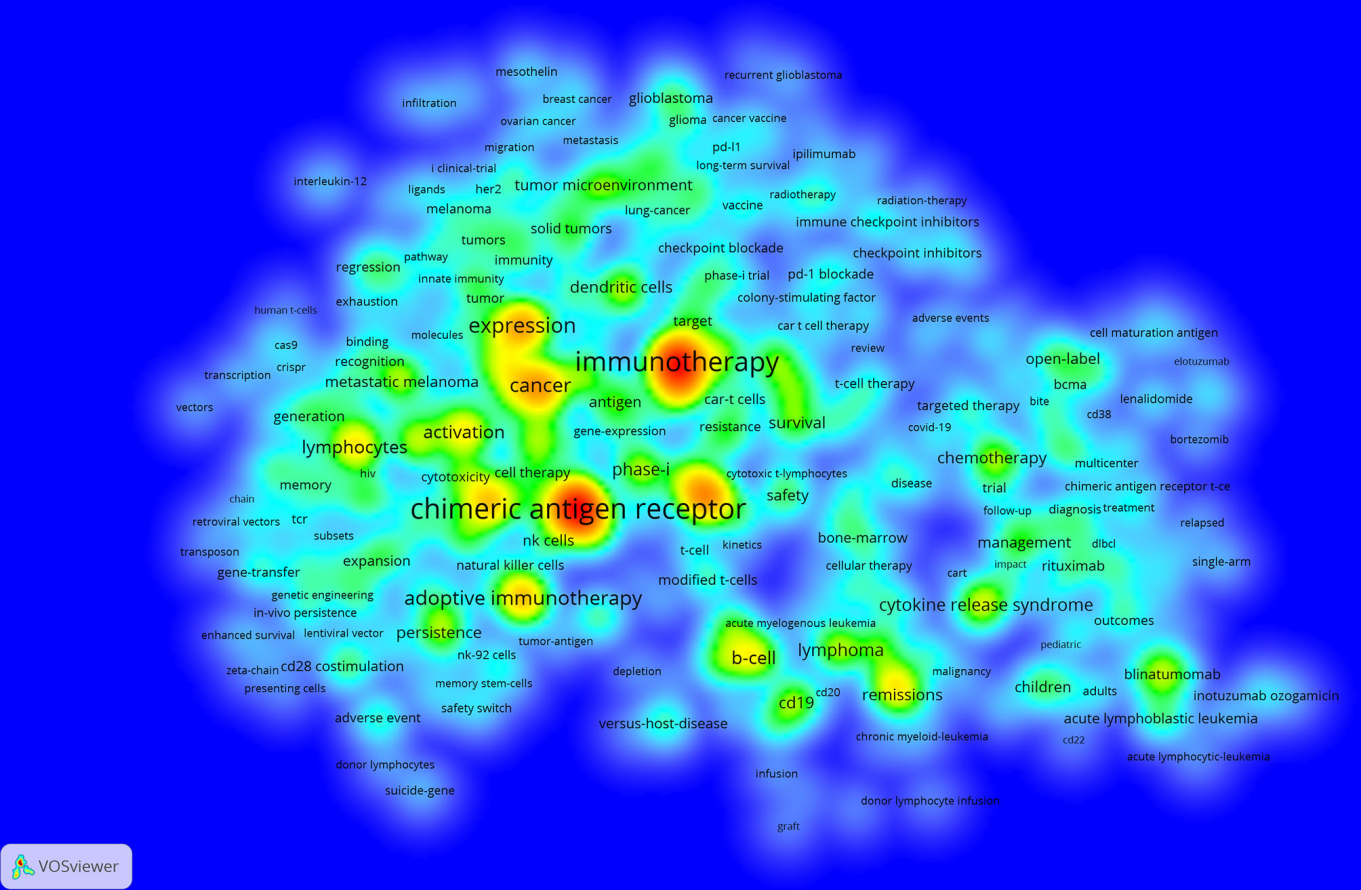
The co-occurrence density map of keywords related to CAR-T cell research. Minimum number of occurrences of keywords ≥20.

We can know the research hotspots and directions in this field through the keyword co-occurrence. We extracted 15,542 keywords with VOSviewer. Since chimeric antigen receptor, chimeric antigen receptors, car, cars, chimeric-antigen-receptor, chimeric antigen receptor (car), and chimeric antigen receptors (cars) all meant the same thing, we merged these words. **Table 4** shows that the top 20 keywords appear more than 300 times. The most frequently occurring keywords are chimeric antigen receptor (n=2244), followed by immunotherapy (n=2008), therapy (n=1054), expression (n=958), cancer (n=831), adoptive immunotherapy (n=820) and anti-tumor activity (n=748). These keywords represent the hotspots of CAR-T cell-related research. The density map of keywords can intuitively display these high-frequency keywords (**Figure 7**).

**Table 4:** The top 20 keywords related to CAR-T cells.

Rank	Keyword	Count	Rank	Keyword	Count
1	chimeric antigen receptor	2244	11	b-cell	526
2	immunotherapy	2008	12	cytokine release syndrome	407
3	therapy	1054	13	acute lymphoblastic-leukemia	404
4	expression	958	14	in-vivo	381
5	cancer	831	15	lymphoma	379
6	adoptive immunotherapy	820	16	phase-I	373
7	antitumor-activity	748	17	natural-killer-cells	364
8	t-cells	721	18	CD19	357
9	activation	570	19	dendritic cells	327
10	lymphocytes	563	20	efficacy	326

**Figure 8:**
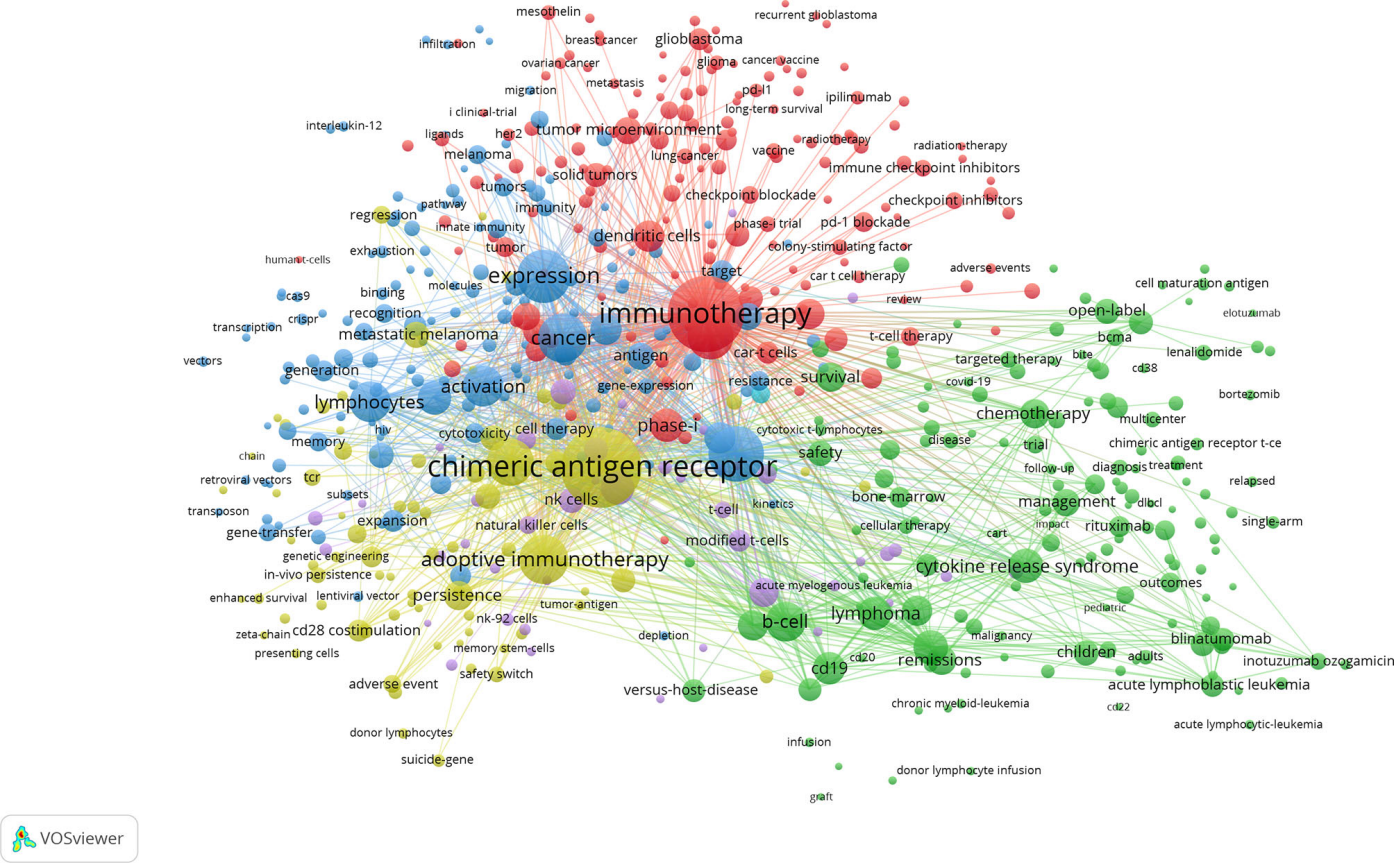
The co-occurrence network and clusters of keywords related to CAR-T cell research. Minimum number of occurrences of keywords ≥20.

We use VOSviewer to perform network clustering analysis on keywords (minimum number of occurrences of a keywors≥20). **Figure 8** shows a total of 6 clusters with different colors obtained, representing 6 research directions and research scopes. The largest cluster is cluster 1 (red), followed by cluster 2 (green), cluster 3 (blue), cluster 4 (yellow), cluster 5 (purple), and cluster 6 (light blue). There are 157 keywords in cluster 1, including immunotherapy, t-cells, phase-I, solid tumor, glioblastoma, breast cancer, lung cancer, growth-factor receptor, immune checkpoint inhibitors, tumor microenvironment, and dendritic cells. There are 152 keywords in cluster 2, including b-cell, CD19, cytokine release syndrome, neurotoxicity, acute lymphoblastic leukemia, lymphoma, multiple myeloma, management, survival, remissions, chemotherapy, and blinatumomab. There are 147 keywords in cluster 3, including therapy, expression, cancer, activation, lymphocytes, gene therapy, *in-vivo*, responses, differentiation, proliferation, memory, resistance, and cytokines. There are 74 keywords in cluster 4, including chimeric antigen receptor, adoptive immunotherapy, anti-tumor activity, anti-tumor efficacy, persistence, regression, adverse event, CD28 costimulation, 4-1BB costimulation, suicide gene, and safety switch. There are 42 keywords in cluster 5, including stem-cells, NK cells, *in-vitro*, messenger-RNA, NK-92 cells, modified-cells, and tumor cells. There is only one keyword (toxicity) in cluster 6.

**Figure 9:**
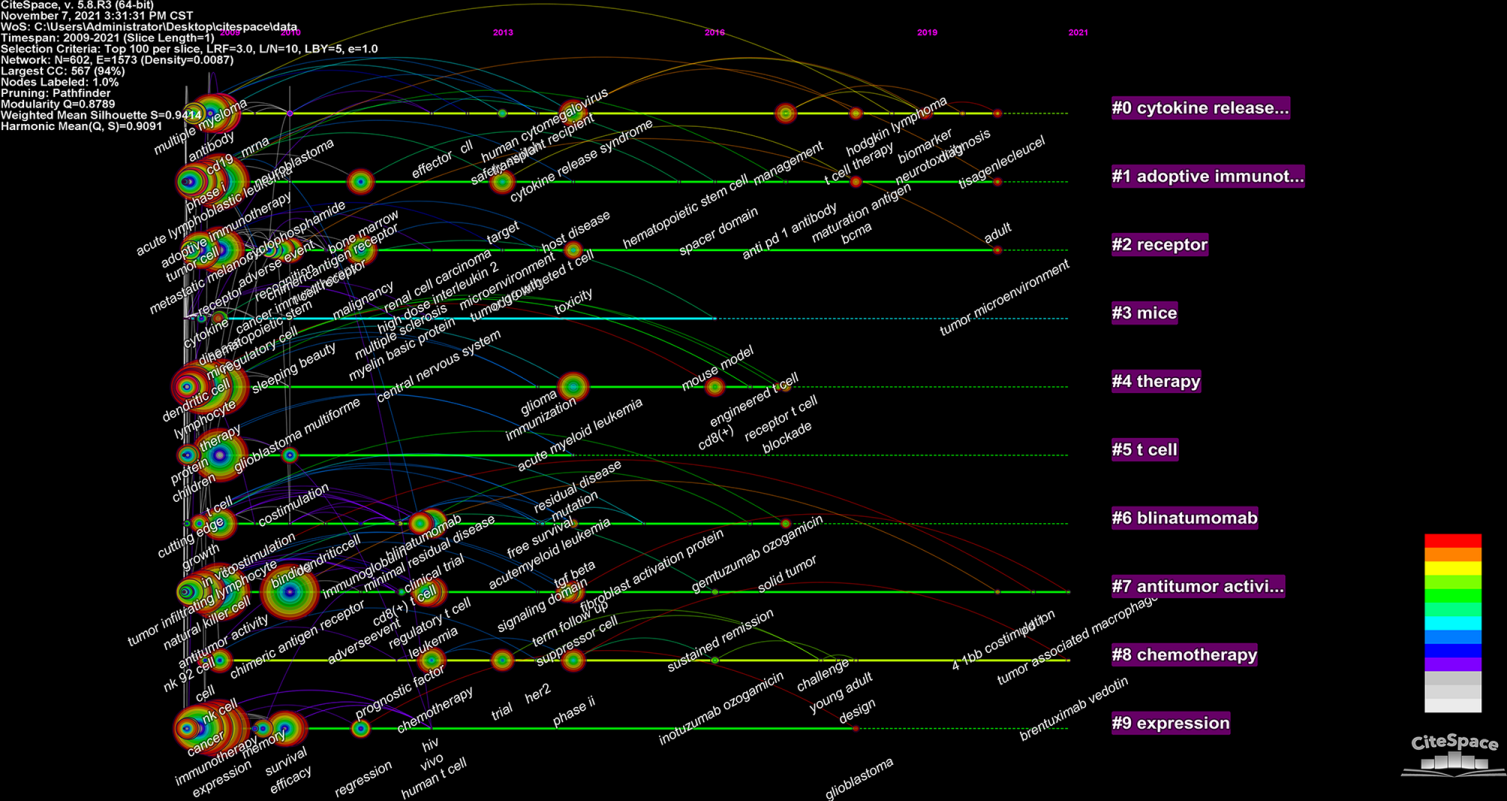
CiteSpace visualization map of timeline viewer related to CAR-T cells.

We used CiteSpace to build a timeline viewer for these keywords. The timeline chart can cluster keywords and take time into account. Therefore, it can show the development of keywords in each cluster. Most importantly, it is convenient for us to see the period of a particular topic in a research field and help us explore the evolution track of this field. From **Figure 9**, we can intuitively see the research focus at each stage and evolution track of CAR-T cells.

### Co-Cited References and Reference Burst

CiteSpace [version 5.8.R3 (64-bit)] was used to find the top 10 most co-cited references. **Table 5** consisted of three parts representing three fields; top 10 co-cited references related to CAR-T cells, top 10 co-cited references related to CAR-T cells for hematological malignancies, and top 10 co-cited references related to CAR-T cells for solid tumors. These 10 references (The first part of **Table 5**) were cited more than 590 times, among which the top 3 references were cited more than 1,000 times. Moreover, the top 3 references were all from *The New England journal of medicine*, and the first authors of the top 2 references were Shannon L Maude. The titles of these two references were “Chimeric antigen receptor T cells for sustained remissions in leukemia” (49) and “Tisagenlecleucel in Children and Young Adults with B-Cell Lymphoblastic Leukemia” (50). It was worth noting that 50% of the top 10 co-cited references were from *The New England Journal of Medicine*. The references in the second part were consistent with the first part, mainly about the study of CD19-CAR-T cells in hematological malignancies. The third part was the top 10 co-cited references related to CAR-T cells for solid tumors (only including solid tumor-related studies). From this part, we could see that 5 of these 10 papers were about the research of CAR-T cells in brain tumors (glioblastoma and neuroblastoma).

**Table 5:** The top 10 co-cited references related to the CAR-T cell field.

Top 10 co-cited references related to CAR-T cells						
Rank	Year	Author	Title	Journal	Citation	Centrality
1	2014	Shannon L Maude et al. (49)	Chimeric antigen receptor T cells for sustained remissions in leukemia	N Engl J Med	1113	0.06
2	2018	Shannon L Maude et al. (50)	Tisagenlecleucel in Children and Young Adults with B-Cell Lymphoblastic Leukemia	N Engl J Med	1065	0.35
3	2017	Sattva S Neelapu et al. (51)	Axicabtagene Ciloleucel CAR T-Cell Therapy in Refractory Large B-Cell Lymphoma	N Engl J Med	1056	0.25
4	2015	Daniel W Lee et al. (52)	T cells expressing CD19 chimeric antigen receptors for acute lymphoblastic leukaemia in children and young adults: a phase 1 dose-escalation trial	Lancet	987	0.47
5	2014	Marco L Davila et al. (53)	Efficacy and toxicity management of 19-28z CAR T cell therapy in B cell acute lymphoblastic leukemia	Sci Transl Med	743	0.54
6	2013	Stephan A Grupp et al. (54)	Chimeric antigen receptor-modified T cells for acute lymphoid leukemia	N Engl J Med	737	0.57
7	2016	Cameron J Turtle et al. (55)	CD19 CAR-T cells of defined CD4+:CD8+ composition in adult B cell ALL patients	J Clin Invest	729	0.03
8	2018	Jae H Park et al. (56)	Long-Term Follow-up of CD19 CAR Therapy in Acute Lymphoblastic Leukemia	N Engl J Med	649	0.35
9	2015	James N Kochenderfer et al. (57)	Chemotherapy-refractory diffuse large B-cell lymphoma and indolent B-cell malignancies can be effectively treated with autologous T cells expressing an anti-CD19 chimeric antigen receptor	J Clin Oncol	626	0.05
10	2013	Renier J Brentjens et al. (58)	CD19-targeted T cells rapidly induce molecular remissions in adults with chemotherapy-refractory acute lymphoblastic leukemia	Sci Transl Med	599	0.00
**Top 10 co-cited references related to CAR-T cells for hematological malignancies**						
**Rank**	**Year**	**Author**	**Title**	**Journal**	**Citation**	**Centrality**
1	2017	Sattva S Neelapu et al. (51)	Axicabtagene Ciloleucel CAR T-Cell Therapy in Refractory Large B-Cell Lymphoma	N Engl J Med	405	0.07
2	2014	Shannon L Maude et al. (49)	Chimeric antigen receptor T cells for sustained remissions in leukemia	N Engl J Med	389	0
3	2018	Shannon L Maude et al. (50)	Tisagenlecleucel in Children and Young Adults with B-Cell Lymphoblastic Leukemia	N Engl J Med	386	0.19
4	2015	Daniel W Lee et al. (52)	T cells expressing CD19 chimeric antigen receptors for acute lymphoblastic leukaemia in children and young adults: a phase 1 dose-escalation trial	Lancet	382	0.23
5	2016	Cameron J Turtle et al. (55)	CD19 CAR-T cells of defined CD4+:CD8+ composition in adult B cell ALL patients	J Clin Invest	291	0.01
6	2014	Marco L Davila et al. (53)	Efficacy and toxicity management of 19-28z CAR T cell therapy in B cell acute lymphoblastic leukemia	Sci Transl Med	265	0.45
7	2019	Stephen J Schuster et al. (59)	Tisagenlecleucel in Adult Relapsed or Refractory Diffuse Large B-Cell Lymphoma	N Engl J Med	260	0.05
8	2018	Jae H Park et al. (56)	Long-Term Follow-up of CD19 CAR Therapy in Acute Lymphoblastic Leukemia	N Engl J Med	256	0.19
9	2013	Stephan A Grupp et al. (54)	Chimeric antigen receptor-modified T cells for acute lymphoid leukemia	N Engl J Med	251	0.13
10	2015	James N Kochenderfer et al. (57)	Chemotherapy-refractory diffuse large B-cell lymphoma and indolent B-cell malignancies can be effectively treated with autologous T cells expressing an anti-CD19 chimeric antigen receptor	J Clin Oncol	233	0.02
**Top 10 co-cited references related to CAR-T cells for solid tumors(only including solid tumor-related studies)**						
**Rank**	**Year**	**Author**	**Title**	**Journal**	**Citation**	**Centrality**
1	2016	Christine E Brown et al. (60)	Regression of Glioblastoma after Chimeric Antigen Receptor T-Cell Therapy	N Engl J Med	134	0.01
2	2017	Donald M O’Rourke et al. (61)	A single dose of peripherally infused EGFRvIII-directed CAR T cells mediates antigen loss and induces adaptive resistance in patients with recurrent glioblastoma	Sci Transl Med	120	0.01
3	2015	Nabil Ahmed et al. (62)	Human Epidermal Growth Factor Receptor 2 (HER2) -Specific Chimeric Antigen Receptor-Modified T Cells for the Immunotherapy of HER2-Positive Sarcoma	J Clin Oncol	90	0.03
4	2017	Nabil Ahmed et al. (63)	HER2-Specific Chimeric Antigen Receptor-Modified Virus-Specific T Cells for Progressive Glioblastoma: A Phase 1 Dose-Escalation Trial	JAMA Oncol	71	0.01
5	2016	Leonid Cherkassky et al. (64)	Human CAR T cells with cell-intrinsic PD-1 checkpoint blockade resist tumor-mediated inhibition	J Clin Invest	68	0.01
6	2010	Richard A Morgan et al. (65)	Case report of a serious adverse event following the administration of T cells transduced with a chimeric antigen receptor recognizing ERBB2	Mol Ther	59	0.09
7	2014	Gregory L Beatty et al. (66)	Mesothelin-specific chimeric antigen receptor mRNA-engineered T cells induce anti-tumor activity in solid malignancies	Cancer Immunol Res	59	0.02
8	2018	Sarwish Rafiq et al. (67)	Targeted delivery of a PD-1-blocking scFv by CAR-T cells enhances anti-tumor efficacy *in vivo*	Nat Biotechnol	48	0.01
9	2011	Chrystal U Louis et al. (68)	Antitumor activity and long-term fate of chimeric antigen receptor-positive T cells in patients with neuroblastoma	Blood	45	0.09
10	2015	Christine E Brown et al. (69)	Bioactivity and Safety of IL13Rα2-Redirected Chimeric Antigen Receptor CD8+ T Cells in Patients with Recurrent Glioblastoma	Clin Cancer Res	43	0

**Figure 10:**
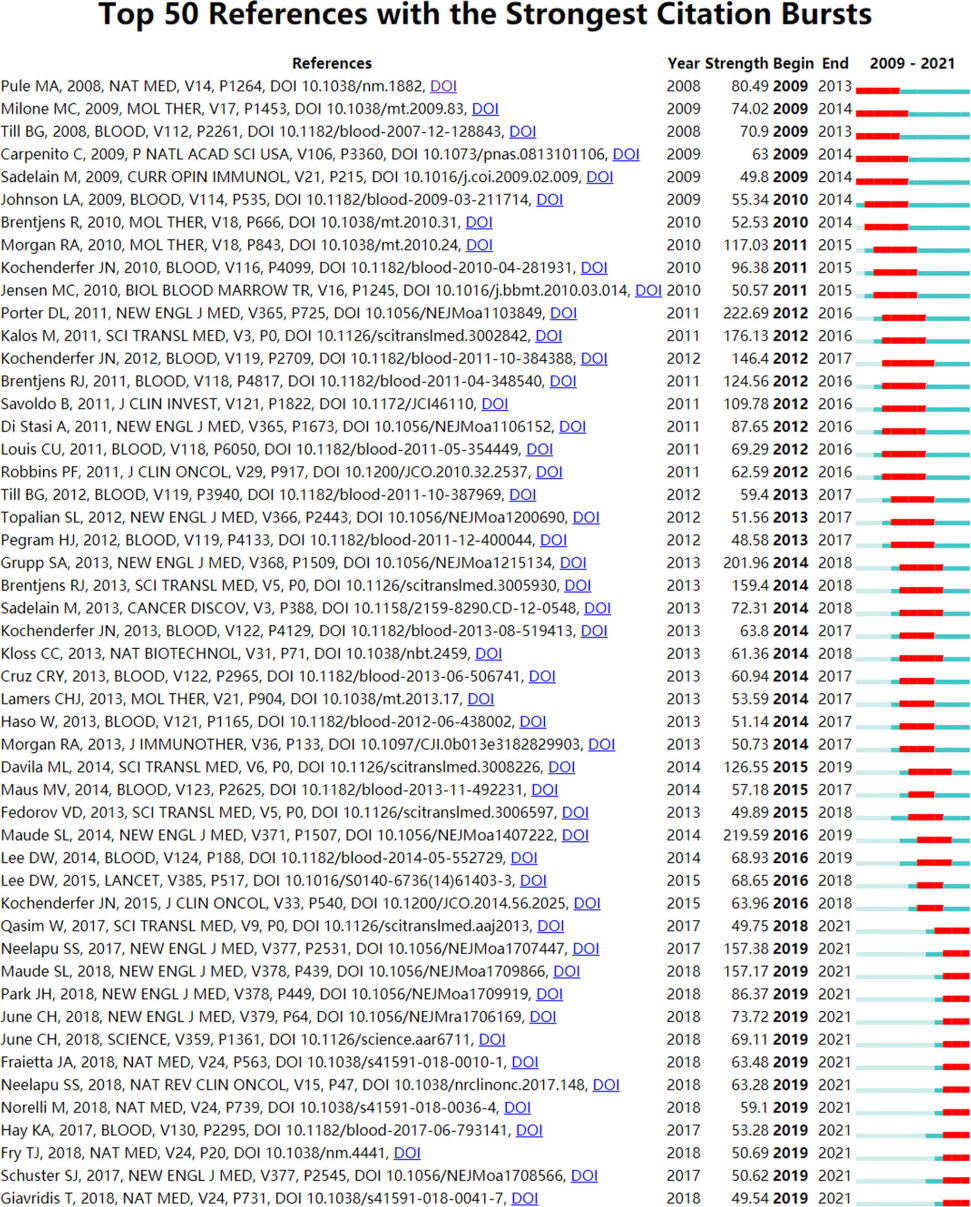
The top 50 references with the strongest citation bursts involved in CAR-T cells. The Blue bars mean the reference had been published; the red bars mean citation burstness.

We used CiteSpace (Selection Criteria: Top50; The Number of States: 2; Minimum Duration: 2) to obtain 157 references with the most robust citation bursts for CAR-T cells. **Figure 10** shows the top 50 among them. The titles of top 3 references with the most vigorous citation bursts were “Chimeric Antigen Receptor-Modified T Cells in Chronic Lymphoid Leukemia (70)” (Strength: 222.69; Publication Year: 2011), “Chimeric Antigen Receptor T Cells for Sustained Remissions in Leukemia (49)” (Strength: 219.59; Publication Year: 2014) and “Chimeric Antigen Receptor–Modified T Cells for Acute Lymphoid Leukemia (54)” (Strength: 201.96; Publication Year: 2013). These three references were all from *The New England Journal of Medicine*. Notably, 13 references (26%; Publication Year: 2017 - 2018) in the Top50 were in a citation burst. The citation bursts of 43 references (86%) were from 2011 to 2021. That is, these references were frequently cited within 10 years. All these mean that CAR-T cell-related research fields may continue to receive attention in the future. In addition, we constructed two additional figures, namely “Top 50 References with the Strongest Citation Bursts for CAR-T cells (hematological malignancies)” **(Annexes 3)** and “Top 50 References with the Strongest Citation Bursts for CAR-T cells (solid tumors)” **(Annexes 4)**. Using these two figures, we drew two corresponding tables **(Annexes 5**, **6)** to introduce these references in the state of citation burst. As seen from **Annexes 3, 5**, there are 11 articles in the 14 papers. These articles are mainly about the research of CAR-T cells in hematological malignancies, including efficacy, safety, mechanism and management of related toxicity, and prognosis. The targets involved in these articles are mainly CD19, followed by CD22. It can be seen from **Annexes 4, 6** that 10 of the 17 articles are about the research of CAR-T cells in hematological malignancies. It shows that the research of CAR-T cells in hematological malignancies greatly influences the research of CAR-T cells in solid tumors. Moreover, 7 articals are about the research of CAR-T cells in solid tumors, which represent some emerging topics in this field. The solid tumors involved in these articles mainly include glioblastoma (related targets: IL13Rα2, EGFRvIII, and HER2), neuroblastoma (related target: GD2), sarcoma (related target: HER2), and pancreatic cancer (related target: mesothelin), especially glioblastoma.

## Discussion

### General Information

Through statistics on CAR-T cell papers published every year, we can understand the development trend of this field. CAR-T cells were first proposed and designed by Israeli scientists Eshhar et al. in 1989. At that time, they called the” “chimeric antigen receptor” as “chimeric T-cell receiver (cTCR)” (71, 72). From **Figure 1**, we can see that the annual growth trend of publication outputs related to CAR-T cells is generally on the rise. It can be divided into three stages, including a continuous period (1990-2009), a slow growth period (2009-2012), and a rapid growth period (2012-2020). From 1990 to 2009, the annual number of publications was minimal, indicating that researchers ignored this field, mainly because the technology related to CAR-T cells is still immature. From 2009 to 2012, papers showed slow growth, indicating that this field received attention. After 2012, the number of papers in this field increased every year, which indicates that this field began to receive extensive attention in this period. Since 2012, the CAR-T cell field entered a period of rapid development, which may be mainly due to three events. The first event was that 7-year-old Emily Whitehead, with acute lymphoblastic leukemia (ALL), became the first child patient in the world to receive CAR-T cell therapy. Encouragingly, Emily got complete remission after receiving CD19-CAR-T cell therapy (54). On December 9th, 2013, the second event was that Science selected the top 10 scientific breakthroughs in 2013, and “cancer immunotherapy” ranked first. The third event was a clinical trial in 2014, in which 30 relapsed ALL patients (children and adults) received CD19-CAR-T cells, and the results showed that 90% of these patients achieved complete remission (49). These three major events aroused scientists’ enthusiasm for CAR-T cell therapy, which greatly promoted the development of this field. Additionally, as of October 28, 1,496 related papers have been published in 2021. There are still two months left before 2022. According to the current trend, publication output in 2021 will likely exceed that in 2020 and continue to grow positively. It proves that the field of CAR-T cells will continue to receive attention in the future.

This field is also very characteristic in the distribution of countries and institutions. From **Table 1** and **Figure 2A**, the country with the most publications in United States (n=3,554, accounting for 51.8% of the total), followed by China (n=1,253, 18.2%) and Germany (n=703, 10.2%). Moreover, the top 10 institutions were all from the United States, and the institution with the largest output of publications is Univ Penn (n=419, 6.10%). Not only that, so far, five CAR-T cell therapies have been approved by Food And Drug Administration (FDA) for marketing in the United States. All these indicate that the United States is the most influential country in CAR-T cells, and the research in this field is far ahead of other countries. China follows it. In recent years, research in this field in China has also increased significantly (73). From **Table 1**, we can also find an interesting phenomenon that 90% of the top 10 countries with the largest publication outputs are developed countries. The main reason for this phenomenon is that the research and development (R&D) of CAR-T cell-related technology needs a large amount of financial support (74). Besides, CAR-T cell therapy is also an expensive treatment. For example, the cost of treating non-Hodgkin lymphoma (NHL) with Kymriah (tisagenlecleucel, CTL019) or Yescarta (axicabtagene ciloleucel, axi-cel) is about $373,000; The cost of Kymriah in treating ALL is approximately $475,000 (75). High R&D costs and clinical use costs limit the clinical promotion and application of this therapy to some extent. Reducing these costs and making the price of CAR-T cell therapy more affordable is also a difficult question that we are facing (76, 77). As shown in **Figure 2A**, the connection between countries is sparse, indicating little cooperation between countries. From **Figure 2B**, we can see that the purple and gray connections are the most, which indicates that the most intensive years of inter-agency cooperation are 2012 and before 2012, and there is little inter-agency cooperation after 2012. We call for strengthening the exchanges and cooperation between countries and institutions in this field to better promote the development of this field and benefit more cancer patients.

Journals and Co-cited Journals Analysis (**Table 2** and **Figure 3**) showed that the journals that published the most CAR-T cell-related papers were *Frontiers in immunology* (n=296) and *Cancers* (n=169). *Blood* (n=46825) and *The New England Journal of Medicine* (n=20112) were frequently co-cited. Among the top 10 journals with published papers, 6 journals were located in the Q1 JCR region, of which the highest IF was Blood (IF=23.629). The top 10 journals with co-cited times were located in the Q1 JCR region. 9 journals had IF greater than 11, and 4 had IF greater than 44. The journal with the highest IF was *The New England Journal of Medicine* (IF=91.253). These indicate that many high-quality and high-impact journals are very interested in CAR-T cell-related research. **Figure 4** shows that papers published in “ Molecular, Biology, Genetics ” journals and “ Health, Nursing, Medicine ” journals are often cited in papers published in “ Molecular, Biology, Immunology ” journals and “ Medicine, medical, Clinical ” journals. It means that the current research related to CAR-T cells mainly focuses on basic research and translational medicine.

In our analysis (**Table 3**, **Figure 5**, and **Figure 6**), Carl H June (n=133) published the most papers, while Shannon L Maude had the most co-citations (n=2446). Among the top 10 co-cited authors, 6 authors played an essential role as a bridge (centrality>0.1). It should be mentioned that the top 10 authors with the most papers had little cooperation with each other; the top 10 authors with the highest number of citations also had little cooperation.

### Knowledge Base

Co-citation is a research method to measure the degree of relevance between papers. The knowledge base is a collection of co-cited references (46). In this study, a total of 10 papers related to the field of CAR-T cells were included, which were co-cited most frequently (the first part of **Table 5**), as follows:

Maude et al. published “Chimeric antigen receptor T cells for sustained remissions in leukemia (49)” in *The New England Journal of Medicine* in 2014, which was the most cited paper (1113 citations). This study was a phase I/IIa clinical trial in which 30 relapsed ALL patients (children and adults) received CD19-CAR-T cells (CTL019). The experimental results showed that 27 patients (90%) achieved complete remission, and 19 observed sustained remission for 2 years. In addition, all patients developed cytokine release syndrome (CRS), and CRS could be effectively improved by toxoximab, an IL-6 receptor inhibitor (49). The second co-cited paper, “Tisagenlecleucel in Children and Young Adults with B-Cell Lymphoblastic Leukemia”, was published by Maude et al. (50) in *The New England Journal of Medicine* in 2018. In this phase II clinical trial, 75 patients with CD19+ relapsed or refractory B-cell all received tisagenlecleucel (Kymriah, the first CAR-T cell therapy in the world). The results showed that (50), the total remission rate of these patients was 81%; the 6-month and 12-month overall survival rate were 90% and 76%, respectively; 55 patients (73%) had a grade 3 or 4 tisagenlecleucel-related adverse event. The third co-cited paper, “Axicabtagene Ciloleucel CAR T-Cell Therapy in Refractory Large B-Cell Lymphoma” was published by Neelapu et al. (51) in *The New England Journal of Medicine* in 2017. In this phase II clinical trial, 101 patients with CD19+ refractory large B-cell lymphoma received axicabotage ciloleucel (Yescarta, the second CAR-T cell therapy in the world). The results showed that (51) the objective response rate (ORR) and complete response rate (CRR) were 82% and 54%, respectively. The 18-month overall survival rate was 52%. The most common adverse events of grade 3 or higher were neutropenia (78%), anemia (43%), and thrombocytopenia (38%). CRS (grade 1~4) occurred in 94 patients (93%) and neurological events (grade 1~4) occurred in 65 patients (64%). The fourth co-cited paper was published by Lee et al. (52) in *Lancet* in 2015. In this phase I clinical trial, 21 patients with CD19+ B-ALL or non-Hodgkin lymphoma (NHL) received CD19-CAR-T cells. The results showed that (52) CD19-CAR-T cells had effective anti-tumor activity and were feasible and safe. The fifth co-cited paper was published by Davila et al. (53) in *Science Translational Medicine* in 2014. This phase I clinical trial mainly evaluated the efficacy of CD19-CAR-T cells in B-All patients (the overall complete remission rate was 88%) and put forward the diagnostic criteria and management scheme of severe cytokine release syndrome (sCRS) (53). The sixth co-cited paper was published by Grupp et al. (54) in *The New England Journal of Medicine* in 2013. This study was a case report. Two children with relapsed and refractory pre-B-cell ALL received CD19-CAR-T cell therapy, and both patients got complete remission. Nevertheless, one of them relapsed two months after receiving treatment (54). The seventh co-cited paper was published by Turtle et al. (55) in *The Journal of Clinical Investigation* in 2016. This phase I clinical trial proved for the first time that it was feasible to select different T cell subsets (CD4+T cells and CD8+T cells) to construct CD19-CAR-T cells (55). The eighth co-cited paper was published by Park et al. (56) in *The New England Journal of Medicine* in 2018. The phase I clinical trial mainly showed the long-term follow-up outcomes of relapsed B-ALL patients who received CD19-CAR-T cell therapy and evaluated the safety of this therapy (56). The ninth co-cited paper was published by Kochenderfer et al. (57) in the *Journal of clinical oncology* in 2015. The phase I clinical trial mainly evaluated the safety and effectiveness of CD19-CAR-T cells in the treatment of advanced CD19+B cell malignancies (57). The tenth co-cited paper was published by Brentjens et al. (58) in *Science Translational Medicine* in 2013. This phase I clinical trial proved that CD19-CAR-T cells had a significant anti-tumor effect in relapsed B-ALL patients (58).

In general, the top 10 most co-cited papers (the first part of **Table 5**) are all clinical experimental studies of CD19-CAR-T cells in treating hematological malignancies. The emphases of these studies mainly include evaluating the efficacy of CD 19-CAR-T cells in patients with hematological malignancies, evaluating the adverse reactions in the treatment process, and how to deal with these adverse reactions. Furthermore, it can also be seen from **Table 5** that the references in the second part are basically consistent with the first part. The third part is the top 10 co-cited references related to CAR-T cells for solid tumors (only including solid tumor-related studies). From this part, we can see that 5 of these 10 papers are about the research of CAR-T cells in brain tumors (glioblastoma and neuroblastoma).

### The Analysis of Hotspots and Emerging Topics

Keywords can reflect the research hotspots and directions in a specific field. From **Table 4**, the top 20 keywords appear more than 300 times. These keywords represent the research hotspots in the field of CAR-T cells. The more representative keywords include chimeric antigen receptor, immunotherapy, cancer, expression, activation, CRS, ALL, lymphoma, phase-I, CD19, anti-tumor activity, efficiency, and NK cells. From these keywords, we can summarize the general situation of CAR-T cell-related fields, including a. CAR-T cell therapy, which is anti-tumor immunotherapy; b. The activation of CAR-T cells and the expression of CARs are important factors for the function of CAR-T cells; c. CRS is a common and most studied adverse reaction of CAR-T cell therapy (78, 79); d. CAR-T cell therapy is widely used in the research and treatment of hematological malignancies (80, 81); e. Presently, most clinical trials related to CAR-T cells are in phase-I (can be verified in clinicaltrials.gov); f. Anti-tumor activity and efficacy are the research emphases in this field (82, 83); g. CD19 is the most commonly used target in treating hematological malignancies (80, 84); h. CAR-NK cell therapy is currently a research hotspot (85, 86).

The density map of these keywords can show the high-frequency keywords in this field more intuitively (**Figure 7**). The network clustering analysis of keywords (totally divided into 6 clusters) can intuitively show this field’s research direction and scope. As shown in **Figure 8**, we get 6 clusters. The keywords of cluster 1 (red) are mainly about the research of CAR-T cells in solid tumors. The keywords of cluster 2 (green) are mainly about the research of CAR-T cells in hematological malignancies. The keywords of cluster 3 (blue) are mainly CAR-T cells’ primary research and mechanism research in tumors. The keywords of cluster 4 (yellow) are mainly related to improving the efficacy and safety of CAR-T cells. The keywords of cluster 5 (purple) may be the study of some immune cells related to CAR-T cells. There is only one keyword, “ toxicity ”, in cluster 6 (light blue). These six clusters represent CAR-T cells’ research focus and scope to some extent. In addition, from this ranking, we can see that CAR-T research in solid tumors has become one of the critical research focuses. Because compared with hematological malignancies, there are more solid tumors and more patients with solid tumors. The timeline viewer (**Figure 9**) of keywords can help us see the time of a topic in this field and help us explore this field’s evolutionary trajectory.

References with intense citation bursts refer to the sudden increase of citations of certain documents in a certain period, which can help us find emerging topics and research topics that have attracted much attention in a certain field (87). This study obtained 157 references with the most powerful citation bursts and selected the top 50 among them (**Figure 10**). The paper (Strength: 222.69) with the strongest citation burstness was a clinical experimental study published by Porter et al. (70) in *The New England Journal of Medicine* in 2011. They developed a second-generation CAR, with CD19 as the target and 4-1BB as the co-stimulatory molecule. This study proved that (70) CD19-CAR-T cells containing the 4-1BB signal domain had strong and durable anti-leukemia activity. More importantly, this study provided an important basis for the ongoing study of CD19-CAR-T cells in the treatment of B-cell tumors. Up to now, 13 papers (26%) in the top 50 are still in a state of citation burst, and the citation burstness of 12 papers has lasted for 3 years (years: 2019-2021). These 13 papers represent the latest research topics related to CAR-T cells. According to a Ranking by burstness strength (from high to low), the No.1 paper (strength: 157.38) was published by Neelapu et al. (51) in *The New England Journal of Medicine* in 2017. The results showed that axicabtagene ciloleucel (Yescarta) had an exciting therapeutic effect on CD19+ refractory large B-cell lymphoma. Furthermore, this study also evaluated the security of Yescarta in detail. The second-ranked paper (strength: 157.17) was published by Maude et al. (50) in *The New England Journal of Medicine* in 2018. The study proved that tisagenlecleucel (Kymriah) produced a high remission rate and lasting remission for CD19+ relapsed or refractory B-cell ALL patients and evaluated the safety of Kymriah in detail. The third-ranked paper (strength: 86.37) was published by Park et al. (56) in *The New England Journal of Medicine* in 2018. This study mainly showed the long-term follow-up outcomes of patients with relapsed B-cell ALL who received CD19-CAR-T cell therapy and evaluated the safety of this therapy. The fourth-ranked paper (strength: 73.72) was published by June et al. (88) in *The New England Journal of Medicine* in 2018. This review introduced chimeric antigen receptor therapy from aspects of immuno-oncology, CAR-T cell-related toxic reactions, cell engineering, and synthetic biology. The fifth-ranked paper (strength: 69.11) was also published by June et al. (89) in *Science* in 2018. This review introduced the opportunities and challenges of CAR-T cells in human cancer treatment. The sixth-ranked paper (strength: 63.48) was published by Fraietta et al. (90) in *Nature Medicine* in 2018. This study explored the clinical response of CD19-CAR-T cells in chronic lymphocytic leukemia (CLL) patients and the determinants and mechanisms related to drug resistance. The seventh-ranked paper (strength: 63.28) was published by Neelapu et al. (91) in *Nature Reviews Clinical Oncology* in 2018. This review mainly introduced CAR-T cell-related toxic reactions’ evaluation and coping strategies. The eighth-ranked (strength: 59.1) was published by Norelli et al. (92) in *Nature Medicine* in 2018. This study mainly explored the mechanism and treatment of CD19-CAR-T cell-related CRS and neurotoxicity. The ninth-ranked paper (strength: 53.28) was published by Hay et al. (93) in *Blood* in 2017. It mainly explored the dynamics, biomarkers, risk factors, and mechanism of severe CRS(sCRS) in CD19-CAR-T cell therapy, and the tenth-ranked paper (strength: 50.69) was published by Fry et al. (94) in *Nature Medicine* in 2018. This study confirmed the clinical activity of CD22-CAR-T cells in CD19-/CD19+ B-ALL for the first time. The eleventh-ranked paper (Strength: 50.62) was published by Schuster et al. (95) in *The New England Journal of Medicine* in 2017. The study mainly explored the efficacy of CD19-CAR-T cells in adult lymphoma. The twelfth-ranked paper (strength: 49.75) was published by Qasim et al. (96) in *Science Translational Medicine* in 2017. Qasim et al. used gene-editing technology to construct CD19-CAR-T cells and at the same time destroyed the TCR and CD52 of these T cells. These special CD19-CAR-T cells (universal CAR19 T cells) could escape the host immunity and reduce the risk of graft-versa-host disease (GVHD). The thirteenth-ranked paper (strength: 49.54) was published by Giavridis et al. (97) in *Nature Medicine* in 2018. This study explored CRS’s mechanism and intervention measures (especially related therapeutic drug “anakinra”) related to CAR-T cells.

According to the above analysis and the related analysis of **Annexes 5, 6**, the following important information can be obtained: a. The high-quality and high-impact research related to CAR-T cells mainly focuses on the clinical experimental studies of CD19-CAR-T cells in hematological malignancies. Related research hotspots include efficacy, safety, drug resistance, and the mechanism and management of related toxic reactions (50, 51, 56, 70, 90, 91); b. CRS is a common and most studied CAR-T cell-related toxic reaction (78, 79). Related studies mainly focus on the mechanism, diagnosis (biomarkers), and intervention (especially related therapeutic drug “anakinra”) of CRS (92, 93, 97); c. CD19 is the most commonly used target of CAR-T cells in treating hematological malignancies (80, 84). Furthermore, CD22 is also a significant target (94); d. Universal CAR-T cells (96) and CAR-NK cells (85, 86) are also the research hotspots; e. The research of CAR-T cells in solid tumors is also a hot field that has developed rapidly. Emerging topics in this field mainly include the study of CAR-T cells in glioblastoma (related targets: IL13Rα2, EGFRvIII, and HER2), neuroblastoma (related target: GD2), sarcoma (related target: HER2), and pancreatic cance  (related target: mesothelin), especially glioblastoma. Furthermore, from the cluster analysis of keywords (**Figure 8**), it can be seen that the research enthusiasm of CAR-T cells in solid tumors can even be compared with that in hematological malignancies.

### Limitation

First of all, this study is a bibliometric study, and CiteSpace and VOSviewer cannot wholly replace system retrieval. Secondly, all data was retrieved and downloaded from the WoSCC database, which would miss some papers not included in this database. However, WoSCC is the most commonly used database in scientific econometric analysis, and the data from WoSCC can represent most information to some extent (98). Finally, because of the large amount of data, CtieSpace ran very slowly in some functions, so we reduced some data (only 12%). All of these may reduce the credibility of this study. Nevertheless, the visual analysis based on reference data can help researchers intuitively understand the CAR-T cell field’s research hotspots, evolution process, and development trend.

## Conclusion

In a word, CAR-T cell therapy is an anti-tumor therapy with great potential and clinical application prospects, which is still in a highly developed stage at present. The related research of CAR-T cells will still be a hot field in the future. The following is a summary of knowledge points and research hotspots in the field of CAR-T cells:

- CAR-T cell-related research is an important research field that many developed countries are interested in, especially the United States is in an absolute leading position. The institution that contributed the most publications is the University of Pennsylvania. However, there is little cooperation between countries. After 2012, cooperation among various institutions is also small;
- The journals that published the most CAR-T cell-related papers are *Frontiers in immunology* and *Cancers*. Nevertheless, *Blood* and *The New England Journal of Medicine* are the most commonly co-cited journals;
- CAR-T cell therapy is a kind of clinical application research. Many high-quality and high-impact journals are very interested in CAR-T cell-related research; especially high-quality clinical trial papers are the most popular;
- Currently, the research related to CAR-T cells mainly focuses on basic research and translational medicine;
- The high-quality and high-impact research related to CAR-T cells mainly focuses on the clinical experimental studies of CD19-CAR-T cells in hematological malignancies. Related research hotspots include efficacy, safety, drug resistance, and the mechanism and management of related toxic reactions;
- CRS is a common and most studied CAR-T cell-related toxic reaction (67, 68). Related studies mainly focus on the mechanism, diagnosis (biomarkers), and intervention (especially related therapeutic drug “anakinra”) of CRS;
- CD19 is the most commonly used target of CAR-T cells in the treatment of hematological malignancies. Furthermore, CD22 is also an important target;
- Universal CAR-T cells (85) and CAR-NK cells (74, 75) are also the research hotspots;
- The research of CAR-T cells in solid tumors is also a hot field that has developed rapidly in recent years. Emerging topics in this field mainly include the study of CAR-T cells in glioblastoma (related targets: IL13Rα2, EGFRvIII, and HER2), neuroblastoma (related target: GD2), sarcoma (related target: HER2), and pancreatic cancer (related target: mesothelin), especially glioblastoma.

## Data Availability Statement

The original contributions presented in the study are included in the article/**Supplementary Material**. Further inquiries can be directed to the corresponding authors.

## Author Contributions

LM: Writing-Original draft preparation, manuscript, investigation, and figure preparation. JZ: manuscript, investigation, and figure preparation. ZZ: Investigation and figure preparation. SW: Investigation. FT: Investigation. MT: Investigation, Methodology, Supervision. YL: Conceptualization, Methodology, Supervision. All authors contributed to the article and approved the submitted version.

## Funding

This work was funded by Special Research Project of Lanzhou University Serving the Economic Social Development of Gansu Province (054000282) Lanzhou Talent Innovation and Entrepreneurship Project (2020-RC-38), the Fundamental Research Funds for the Central Universities (lzujbky-2020-kb14), and Major Science and Technology Special Project of Gansu Province (20ZD7FA003).

## Conflict of Interest

The authors declare that the research was conducted in the absence of any commercial or financial relationships that could be construed as a potential conflict of interest.

## Publisher’s Note

All claims expressed in this article are solely those of the authors and do not necessarily represent those of their affiliated organizations, or those of the publisher, the editors and the reviewers. Any product that may be evaluated in this article, or claim that may be made by its manufacturer, is not guaranteed or endorsed by the publisher.
