# Supplementary material for: A Bibliometric and Knowledge-Map Analysis of CAR-T Cells From 2009 to 2021
Source: Front Immunol. 2022 Mar 18;13:840956. doi: 10.3389/fimmu.2022.840956 (PMC8971369; doi:10.3389/fimmu.2022.840956)
Supplement: Supplementary file 3 [file DataSheet_3.docx]

| **NO.** | **Year** | **Author** | **Article Type** | **Target** | **Associated Tumor** | **Title** | **Strength** |
| --- | --- | --- | --- | --- | --- | --- | --- |
| 1 | 2017 | Turtle *et al*. (1) | article | CD19 | chronic lymphocytic leukemia | Durable Molecular Remissions in Chronic Lymphocytic Leukemia Treated With CD19-Specific Chimeric Antigen Receptor-Modified T Cells After Failure of Ibrutinib | 15.7 |
| 2 | 2017 | Neelapu *et al*. (2) | article | CD19 | diffuse large B-cell lymphoma | Axicabtagene Ciloleucel CAR T-Cell Therapy in Refractory Large B-Cell Lymphoma | 58.89 |
| 3 | 2018 | Maude *et al*. (3) | article | CD19 | acute lymphoblastic leukemia | Tisagenlecleucel in Children and Young Adults with B-Cell Lymphoblastic Leukemia | 51.89 |
| 4 | 2018 | Park *et al*. (4) | article | CD19 | acute lymphoblastic leukemia | Long-Term Follow-up of CD19 CAR Therapy in Acute Lymphoblastic Leukemia | 33.81 |
| 5 | 2018 | Neelapu *et al*. (5) | review |  |  | Chimeric antigen receptor T-cell therapy - assessment and management of toxicities | 20.6 |
| 6 | 2017 | Crump *et al*. (6) | article |  | diffuse large B-cell lymphoma | Outcomes in refractory diffuse large B-cell lymphoma: results from the international SCHOLAR-1 study | 20.32 |
| 7 | 2018 | June *et al*. (7) | review |  |  | Chimeric Antigen Receptor Therapy | 19.5 |
| 8 | 2017 | Schuster *et al*. (8) | article | CD19 | diffuse large B-cell lymphoma; follicular lymphoma | Chimeric Antigen Receptor T Cells in Refractory B-Cell Lymphomas | 17 |
| 9 | 2018 | Norelli *et al*. (9) | article |  |  | Monocyte-derived IL-1 and IL-6 are differentially required for cytokine-release syndrome and neurotoxicity due to CAR T cells | 16.26 |
| 10 | 2018 | Fraietta *et al*. (10) | article | CD19 | chronic lymphocytic leukemia | Determinants of response and resistance to CD19 chimeric antigen receptor (CAR) T cell therapy of chronic lymphocytic leukemia | 16.26 |
| 11 | 2017 | Gardner *et al*. (11) | article | CD19 | acute lymphoblastic leukemia | Intent-to-treat leukemia remission by CD19 CAR T cells of defined formulation and dose in children and young adults | 16.17 |
| 12 | 2018 | Giavridis *et al*. (12) | article | CD19 |  | CAR T cell–induced cytokine release syndrome is mediated by macrophages and abated by IL-1 blockade | 14.64 |
| 13 | 2018 | June *et al*. (13) | review |  |  | CAR T cell immunotherapy for human cancer | 14.64 |
| 14 | 2018 | Fry *et al*. (14) | article | CD22 | acute lymphoblastic leukemia | CD22-targeted CAR T cells induce remission in B-ALL that is naive or resistant to CD19-targeted CAR immunotherapy | 14.26 |

**References:**

1. Turtle CJ, Hay KA, Hanafi LA, Li D, Cherian S, Chen X, et al. Durable Molecular Remissions in Chronic Lymphocytic Leukemia Treated With CD19-Specific Chimeric Antigen Receptor-Modified T Cells After Failure of Ibrutinib. *Journal of clinical oncology : official journal of the American Society of Clinical Oncology* (2017) 35(26):3010-20. Epub 2017/07/18. doi: 10.1200/jco.2017.72.8519.

2. Neelapu SS, Locke FL, Bartlett NL, Lekakis LJ, Miklos DB, Jacobson CA, et al. Axicabtagene Ciloleucel CAR T-Cell Therapy in Refractory Large B-Cell Lymphoma. *The New England journal of medicine* (2017) 377(26):2531-44. Epub 2017/12/12. doi: 10.1056/NEJMoa1707447.

3. Maude SL, Laetsch TW, Buechner J, Rives S, Boyer M, Bittencourt H, et al. Tisagenlecleucel in Children and Young Adults with B-Cell Lymphoblastic Leukemia. *The New England journal of medicine* (2018) 378(5):439-48. Epub 2018/02/01. doi: 10.1056/NEJMoa1709866.

4. Park JH, Rivière I, Gonen M, Wang X, Sénéchal B, Curran KJ, et al. Long-Term Follow-up of CD19 CAR Therapy in Acute Lymphoblastic Leukemia. *The New England journal of medicine* (2018) 378(5):449-59. Epub 2018/02/01. doi: 10.1056/NEJMoa1709919.

5. Neelapu SS, Tummala S, Kebriaei P, Wierda W, Gutierrez C, Locke FL, et al. Chimeric antigen receptor T-cell therapy - assessment and management of toxicities. *Nature reviews Clinical oncology* (2018) 15(1):47-62. Epub 2017/09/20. doi: 10.1038/nrclinonc.2017.148.

6. Crump M, Neelapu SS, Farooq U, Van Den Neste E, Kuruvilla J, Westin J, et al. Outcomes in refractory diffuse large B-cell lymphoma: results from the international SCHOLAR-1 study. *Blood* (2017) 130(16):1800-8. Epub 2017/08/05. doi: 10.1182/blood-2017-03-769620.

7. June CH, Sadelain M. Chimeric Antigen Receptor Therapy. *The New England journal of medicine* (2018) 379(1):64-73. Epub 2018/07/05. doi: 10.1056/NEJMra1706169.

8. Schuster SJ, Svoboda J, Chong EA, Nasta SD, Mato AR, Anak Ö, et al. Chimeric Antigen Receptor T Cells in Refractory B-Cell Lymphomas. *The New England journal of medicine* (2017) 377(26):2545-54. Epub 2017/12/12. doi: 10.1056/NEJMoa1708566.

9. Norelli M, Camisa B, Barbiera G, Falcone L, Purevdorj A, Genua M, et al. Monocyte-derived IL-1 and IL-6 are differentially required for cytokine-release syndrome and neurotoxicity due to CAR T cells. *Nature medicine* (2018) 24(6):739-48. Epub 2018/05/29. doi: 10.1038/s41591-018-0036-4.

10. Fraietta JA, Lacey SF, Orlando EJ, Pruteanu-Malinici I, Gohil M, Lundh S, et al. Determinants of response and resistance to CD19 chimeric antigen receptor (CAR) T cell therapy of chronic lymphocytic leukemia. *Nature medicine* (2018) 24(5):563-71. Epub 2018/05/02. doi: 10.1038/s41591-018-0010-1.

11. Gardner RA, Finney O, Annesley C, Brakke H, Summers C, Leger K, et al. Intent-to-treat leukemia remission by CD19 CAR T cells of defined formulation and dose in children and young adults. *Blood* (2017) 129(25):3322-31. Epub 2017/04/15. doi: 10.1182/blood-2017-02-769208.

12. Giavridis T, van der Stegen SJC, Eyquem J, Hamieh M, Piersigilli A, Sadelain M. CAR T cell-induced cytokine release syndrome is mediated by macrophages and abated by IL-1 blockade. *Nature medicine* (2018) 24(6):731-8. Epub 2018/05/29. doi: 10.1038/s41591-018-0041-7.

13. June CH, O'Connor RS, Kawalekar OU, Ghassemi S, Milone MC. CAR T cell immunotherapy for human cancer. *Science (New York, NY)* (2018) 359(6382):1361-5. Epub 2018/03/24. doi: 10.1126/science.aar6711.

14. Fry TJ, Shah NN, Orentas RJ, Stetler-Stevenson M, Yuan CM, Ramakrishna S, et al. CD22-targeted CAR T cells induce remission in B-ALL that is naive or resistant to CD19-targeted CAR immunotherapy. *Nature medicine* (2018) 24(1):20-8. Epub 2017/11/21. doi: 10.1038/nm.4441.
