# Supplementary material for: A Bibliometric and Knowledge-Map Analysis of CAR-T Cells From 2009 to 2021
Source: Front Immunol. 2022 Mar 18;13:840956. doi: 10.3389/fimmu.2022.840956 (PMC8971369; doi:10.3389/fimmu.2022.840956)
Supplement: Supplementary file 4 [file DataSheet_4.pdf]

Top 50 References with the Strongest Citation Bursts

References	Year	Strength	Begin	End	2009 - 2021
Till BG, 2008, BLOOD, V112, P2261, DOI 10.1182/blood-2007-12-128843, DOI	2008	12.73	**2009**	2013	
Pule MA, 2008, NAT MED, V14, P1264, DOI 10.1038/nm.1882, DOI	2008	19	**2010**	2013	
Carpenito C, 2009, P NATL ACAD SCI USA, V106, P3360, DOI 10.1073/pnas.0813101106, DOI	2009	16.51	**2010**	2014	
Johnson LA, 2009, BLOOD, V114, P535, DOI 10.1182/blood-2009-03-211714, DOI	2009	14.9	**2010**	2014	
Milone MC, 2009, MOL THER, V17, P1453, DOI 10.1038/mt.2009.83, DOI	2009	13.56	**2010**	2014	
Morgan RA, 2010, MOL THER, V18, P843, DOI 10.1038/mt.2010.24, DOI	2010	23.4	**2011**	2015	
Porter DL, 2011, NEW ENGL J MED, V365, P725, DOI 10.1056/NEJMoa1103849, DOI	2011	45.42	**2012**	2016	
Kalos M, 2011, SCI TRANSL MED, V3, P0, DOI 10.1126/scitranslmed.3002842, DOI	2011	30.12	**2012**	2016	
Louis CU, 2011, BLOOD, V118, P6050, DOI 10.1182/blood-2011-05-354449, DOI	2011	17.83	**2012**	2016	
Di Stasi A, 2011, NEW ENGL J MED, V365, P1673, DOI 10.1056/NEJMoa1106152, DOI	2011	16.63	**2012**	2016	
Kochenderfer JN, 2010, BLOOD, V116, P4099, DOI 10.1182/blood-2010-04-281931, DOI	2010	14.7	**2012**	2015	
Savoldo B, 2011, J CLIN INVEST, V121, P1822, DOI 10.1172/JCI46110, DOI	2011	14.64	**2012**	2016	
Robbins PF, 2011, J CLIN ONCOL, V29, P917, DOI 10.1200/JCO.2010.32.2537, DOI	2011	14.64	**2012**	2016	
Rosenberg SA, 2011, CLIN CANCER RES, V17, P4550, DOI 10.1158/1078-0432.CCR-11-0116, DOI	2011	11.85	**2012**	2016	
Kochenderfer JN, 2012, BLOOD, V119, P2709, DOI 10.1182/blood-2011-10-384388, DOI	2012	23.28	**2013**	2017	
Topalian SL, 2012, NEW ENGL J MED, V366, P2443, DOI 10.1056/NEJMoa1200690, DOI	2012	11.92	**2013**	2017	
Grupp SA, 2013, NEW ENGL J MED, V368, P1509, DOI 10.1056/NEJMoa1215134, DOI	2013	35.92	**2014**	2018	
Brentjens RJ, 2013, SCI TRANSL MED, V5, P0, DOI 10.1126/scitranslmed.3005930, DOI	2013	31	**2014**	2018	
Sadelain M, 2013, CANCER DISCOV, V3, P388, DOI 10.1158/2159-8290.CD-12-0548, DOI	2013	15.03	**2014**	2018	
Davila ML, 2014, SCI TRANSL MED, V6, P0, DOI 10.1126/scitranslmed.3008226, DOI	2014	24.43	**2015**	2019	
Beatty GL, 2014, CANCER IMMUNOL RES, V2, P112, DOI 10.1158/2326-6066.CIR-13-0170, DOI	2014	13.84	**2015**	2019	
John LB, 2013, CLIN CANCER RES, V19, P5636, DOI 10.1158/1078-0432.CCR-13-0458, DOI	2013	12.88	**2015**	2018	
Maude SL, 2014, NEW ENGL J MED, V371, P1507, DOI 10.1056/NEJMoa1407222, DOI	2014	49.05	**2016**	2019	
Lee DW, 2015, LANCET, V385, P517, DOI 10.1016/S0140-6736(14)61403-3, DOI	2015	22.66	**2016**	2019	
Kochenderfer JN, 2015, J CLIN ONCOL, V33, P540, DOI 10.1200/JCO.2014.56.2025, DOI	2015	19.35	**2016**	2019	
Porter DL, 2015, SCI TRANSL MED, V7, P0, DOI 10.1126/scitranslmed.aac5415, DOI	2015	14.93	**2016**	2019	
Rosenberg SA, 2015, SCIENCE, V348, P62, DOI 10.1126/science.aaa4967, DOI	2015	14.7	**2016**	2019	
Johnson LA, 2015, SCI TRANSL MED, V7, P0, DOI 10.1126/scitranslmed.aaa4963, DOI	2015	12.33	**2016**	2019	
Ahmed N, 2015, J CLIN ONCOL, V33, P1688, DOI 10.1200/JCO.2014.58.0225, DOI	2015	20.09	**2017**	2021	
Long AH, 2015, NAT MED, V21, P581, DOI 10.1038/nm.3838, DOI	2015	18.47	**2017**	2021	
Turtle CJ, 2016, J CLIN INVEST, V126, P2123, DOI 10.1172/JCI85309, DOI	2016	11.76	**2017**	2021	
Brown CE, 2015, CLIN CANCER RES, V21, P4062, DOI 10.1158/1078-0432.CCR-15-0428, DOI	2015	11.51	**2017**	2021	
ORourke DM, 2017, SCI TRANSL MED, V9, P0, DOI 10.1126/scitranslmed.aaa0984, DOI	2017	29.14	**2018**	2021	
Brown CE, 2016, NEW ENGL J MED, V375, P2561, DOI 10.1056/NEJMoa1610497, DOI	2016	24.07	**2018**	2021	
Ahmed N, 2017, JAMA ONCOL, V3, P1094, DOI 10.1001/jamaoncol.2017.0184, DOI	2017	17.07	**2018**	2021	
Lim WA, 2017, CELL, V168, P724, DOI 10.1016/j.cell.2017.01.016, DOI	2017	14.15	**2018**	2021	
Fesnak AD, 2016, NAT REV CANCER, V16, P566, DOI 10.1038/nrc.2016.97, DOI	2016	12.22	**2018**	2021	
Maude SL, 2018, NEW ENGL J MED, V378, P439, DOI 10.1056/NEJMoa1709866, DOI	2018	35.58	**2019**	2021	
Neelapu SS, 2017, NEW ENGL J MED, V377, P2531, DOI 10.1056/NEJMoa1707447, DOI	2017	30.82	**2019**	2021	
Newick K, 2017, ANNU REV MED, V68, P139, DOI 10.1146/annurev-med-062315-120245, DOI	2017	23.56	**2019**	2021	
June CH, 2018, SCIENCE, V359, P1361, DOI 10.1126/science.aar6711, DOI	2018	21.39	**2019**	2021	
June CH, 2018, NEW ENGL J MED, V379, P64, DOI 10.1056/NEJMra1706169, DOI	2018	18.51	**2019**	2021	
Park JH, 2018, NEW ENGL J MED, V378, P449, DOI 10.1056/NEJMoa1709919, DOI	2018	18.15	**2019**	2021	
Rafiq S, 2018, NAT BIOTECHNOL, V36, P847, DOI 10.1038/nbt.4195, DOI	2018	17.07	**2019**	2021	
Adachi K, 2018, NAT BIOTECHNOL, V36, P346, DOI 10.1038/nbt.4086, DOI	2018	14.2	**2019**	2021	
Schuster SJ, 2017, NEW ENGL J MED, V377, P2545, DOI 10.1056/NEJMoa1708566, DOI	2017	13.94	**2019**	2021	
Beatty GL, 2018, GASTROENTEROLOGY, V155, P29, DOI 10.1053/j.gastro.2018.03.029, DOI	2018	13.84	**2019**	2021	
Roybal KT, 2016, CELL, V164, P770, DOI 10.1016/j.cell.2016.01.011, DOI	2016	12.41	**2019**	2021	
Heczey A, 2017, MOL THER, V25, P2214, DOI 10.1016/j.ymthe.2017.05.012, DOI	2017	12.41	**2019**	2021	
Fry TJ, 2018, NAT MED, V24, P20, DOI 10.1038/nm.4441, DOI	2018	11.34	**2019**	2021	
