# Supplementary material for: A Bibliometric and Knowledge-Map Analysis of CAR-T Cells From 2009 to 2021
Source: Front Immunol. 2022 Mar 18;13:840956. doi: 10.3389/fimmu.2022.840956 (PMC8971369; doi:10.3389/fimmu.2022.840956)
Supplement: Supplementary file 5 [file DataSheet_5.docx]

| **NO.** | **Year** | **Author** | **Article Type** | **Target** | **Associated Tumor** | **Title** | **Strength** |
| --- | --- | --- | --- | --- | --- | --- | --- |
| 1 | 2017 | Turtle et al. (1) | article | CD19 | chronic lymphocytic leukemia | Durable Molecular Remissions in Chronic Lymphocytic Leukemia Treated With CD19-Specific Chimeric Antigen Receptor-Modified T Cells After Failure of Ibrutinib | 15.7 |
| 2 | 2017 | Neelapu et al. (2) | article | CD19 | diffuse large B-cell lymphoma | Axicabtagene Ciloleucel CAR T-Cell Therapy in Refractory Large B-Cell Lymphoma | 58.89 |
| 3 | 2018 | Maude et al. (3) | article | CD19 | acute lymphoblastic leukemia | Tisagenlecleucel in Children and Young Adults with B-Cell Lymphoblastic Leukemia | 51.89 |
| 4 | 2018 | Park et al. (4) | article | CD19 | acute lymphoblastic leukemia | Long-Term Follow-up of CD19 CAR Therapy in Acute Lymphoblastic Leukemia | 33.81 |
| 5 | 2018 | Neelapu et al. (5) | review |  |  | Chimeric antigen receptor T-cell therapy - assessment and management of toxicities | 20.6 |
| 6 | 2017 | Crump et al. (6) | article |  | diffuse large B-cell lymphoma | Outcomes in refractory diffuse large B-cell lymphoma: results from the international SCHOLAR-1 study | 20.32 |
| 7 | 2018 | June et al. (7) | review |  |  | Chimeric Antigen Receptor Therapy | 19.5 |
| 8 | 2017 | Schuster et al. (8) | article | CD19 | diffuse large B-cell lymphoma; follicular lymphoma | Chimeric Antigen Receptor T Cells in Refractory B-Cell Lymphomas | 17 |
| 9 | 2018 | Norelli et al. (9) | article |  |  | Monocyte-derived IL-1 and IL-6 are differentially required for cytokine-release syndrome and neurotoxicity due to CAR T cells | 16.26 |
| 10 | 2018 | Fraietta et al. (10) | article | CD19 | chronic lymphocytic leukemia | Determinants of response and resistance to CD19 chimeric antigen receptor (CAR) T cell therapy of chronic lymphocytic leukemia | 16.26 |
| 11 | 2017 | Gardner et al. (11) | article | CD19 | acute lymphoblastic leukemia | Intent-to-treat leukemia remission by CD19 CAR T cells of defined formulation and dose in children and young adults | 16.17 |
| 12 | 2018 | Giavridis et al. (12) | article | CD19 |  | CAR T cell–induced cytokine release syndrome is mediated by macrophages and abated by IL-1 blockade | 14.64 |
| 13 | 2018 | June et al. (13) | review |  |  | CAR T cell immunotherapy for human cancer | 14.64 |
| 14 | 2018 | Fry et al. (14) | article | CD22 | acute lymphoblastic leukemia | CD22-targeted CAR T cells induce remission in B-ALL that is naive or resistant to CD19-targeted CAR immunotherapy | 14.26 |

**References:**

1. Turtle CJ, Hay KA, Hanafi LA, Li D, Cherian S, Chen X, et al. Durable Molecular Remissions in Chronic Lymphocytic Leukemia Treated With CD19-Specific Chimeric Antigen Receptor-Modified T Cells After Failure of Ibrutinib. *Journal of clinical oncology : official journal of the American Society of Clinical Oncology* (2017) 35(26):3010-20. Epub 2017/07/18. doi: 10.1200/jco.2017.72.8519. PubMed PMID: 28715249; PubMed Central PMCID: PMCPMC5590803.

2. Neelapu SS, Locke FL, Bartlett NL, Lekakis LJ, Miklos DB, Jacobson CA, et al. Axicabtagene Ciloleucel CAR T-Cell Therapy in Refractory Large B-Cell Lymphoma. *The New England journal of medicine* (2017) 377(26):2531-44. Epub 2017/12/12. doi: 10.1056/NEJMoa1707447. PubMed PMID: 29226797; PubMed Central PMCID: PMCPMC5882485.

3. Maude SL, Laetsch TW, Buechner J, Rives S, Boyer M, Bittencourt H, et al. Tisagenlecleucel in Children and Young Adults with B-Cell Lymphoblastic Leukemia. *The New England journal of medicine* (2018) 378(5):439-48. Epub 2018/02/01. doi: 10.1056/NEJMoa1709866. PubMed PMID: 29385370; PubMed Central PMCID: PMCPMC5996391.

4. Park JH, Rivière I, Gonen M, Wang X, Sénéchal B, Curran KJ, et al. Long-Term Follow-up of CD19 CAR Therapy in Acute Lymphoblastic Leukemia. *The New England journal of medicine* (2018) 378(5):449-59. Epub 2018/02/01. doi: 10.1056/NEJMoa1709919. PubMed PMID: 29385376; PubMed Central PMCID: PMCPMC6637939.

5. Neelapu SS, Tummala S, Kebriaei P, Wierda W, Gutierrez C, Locke FL, et al. Chimeric antigen receptor T-cell therapy - assessment and management of toxicities. *Nature reviews Clinical oncology* (2018) 15(1):47-62. Epub 2017/09/20. doi: 10.1038/nrclinonc.2017.148. PubMed PMID: 28925994; PubMed Central PMCID: PMCPMC6733403.

6. Crump M, Neelapu SS, Farooq U, Van Den Neste E, Kuruvilla J, Westin J, et al. Outcomes in refractory diffuse large B-cell lymphoma: results from the international SCHOLAR-1 study. *Blood* (2017) 130(16):1800-8. Epub 2017/08/05. doi: 10.1182/blood-2017-03-769620. PubMed PMID: 28774879; PubMed Central PMCID: PMCPMC5649550 a consultant and an advisory board member for Kite Pharma. U.F. received research funding from Kite Pharma. J.K. received research funding from Celgene, Roche, and Karyopharm; served as a consultant for Bristol-Myers Squibb, Gilead, Janssen, Hoffman LaRoche, and Seattle Genetics; and received honoraria from Amgen, Bristol-Myers Squibb, Celgene, Gilead, Roche, Janssen, Lundbeck, Merck, and Seattle Genetics. J.W. served on advisory boards for ProNAi Therapeutics, Celgene, and Genentech and received research funding from Janssen, Celgene, Genentech, Kite, and Novartis. B.K.L., J.R.C., and M.J.M. received research funding from Kite Pharma. L.N., J.W., and W.Y.G. are employed by and have equity ownership in Kite Pharma. The remaining authors declare no competing financial interests.

7. June CH, Sadelain M. Chimeric Antigen Receptor Therapy. *The New England journal of medicine* (2018) 379(1):64-73. Epub 2018/07/05. doi: 10.1056/NEJMra1706169. PubMed PMID: 29972754; PubMed Central PMCID: PMCPMC7433347.

8. Schuster SJ, Svoboda J, Chong EA, Nasta SD, Mato AR, Anak Ö, et al. Chimeric Antigen Receptor T Cells in Refractory B-Cell Lymphomas. *The New England journal of medicine* (2017) 377(26):2545-54. Epub 2017/12/12. doi: 10.1056/NEJMoa1708566. PubMed PMID: 29226764; PubMed Central PMCID: PMCPMC5788566.

9. Norelli M, Camisa B, Barbiera G, Falcone L, Purevdorj A, Genua M, et al. Monocyte-derived IL-1 and IL-6 are differentially required for cytokine-release syndrome and neurotoxicity due to CAR T cells. *Nature medicine* (2018) 24(6):739-48. Epub 2018/05/29. doi: 10.1038/s41591-018-0036-4. PubMed PMID: 29808007.

10. Fraietta JA, Lacey SF, Orlando EJ, Pruteanu-Malinici I, Gohil M, Lundh S, et al. Determinants of response and resistance to CD19 chimeric antigen receptor (CAR) T cell therapy of chronic lymphocytic leukemia. *Nature medicine* (2018) 24(5):563-71. Epub 2018/05/02. doi: 10.1038/s41591-018-0010-1. PubMed PMID: 29713085; PubMed Central PMCID: PMCPMC6117613.

11. Gardner RA, Finney O, Annesley C, Brakke H, Summers C, Leger K, et al. Intent-to-treat leukemia remission by CD19 CAR T cells of defined formulation and dose in children and young adults. *Blood* (2017) 129(25):3322-31. Epub 2017/04/15. doi: 10.1182/blood-2017-02-769208. PubMed PMID: 28408462; PubMed Central PMCID: PMCPMC5482103.

12. Giavridis T, van der Stegen SJC, Eyquem J, Hamieh M, Piersigilli A, Sadelain M. CAR T cell-induced cytokine release syndrome is mediated by macrophages and abated by IL-1 blockade. *Nature medicine* (2018) 24(6):731-8. Epub 2018/05/29. doi: 10.1038/s41591-018-0041-7. PubMed PMID: 29808005; PubMed Central PMCID: PMCPMC6410714.

13. June CH, O'Connor RS, Kawalekar OU, Ghassemi S, Milone MC. CAR T cell immunotherapy for human cancer. *Science (New York, NY)* (2018) 359(6382):1361-5. Epub 2018/03/24. doi: 10.1126/science.aar6711. PubMed PMID: 29567707.

14. Fry TJ, Shah NN, Orentas RJ, Stetler-Stevenson M, Yuan CM, Ramakrishna S, et al. CD22-targeted CAR T cells induce remission in B-ALL that is naive or resistant to CD19-targeted CAR immunotherapy. *Nature medicine* (2018) 24(1):20-8. Epub 2017/11/21. doi: 10.1038/nm.4441. PubMed PMID: 29155426; PubMed Central PMCID: PMCPMC5774642.
