# Supplementary material for: A Bibliometric and Knowledge-Map Analysis of CAR-T Cells From 2009 to 2021
Source: Front Immunol. 2022 Mar 18;13:840956. doi: 10.3389/fimmu.2022.840956 (PMC8971369; doi:10.3389/fimmu.2022.840956)
Supplement: Supplementary file 6 [file DataSheet_6.docx]

| **NO.** | **Year** | **Author** | **Article Type** | **Target** | **Associated Tumor** | **Title** | **Strength** |
| --- | --- | --- | --- | --- | --- | --- | --- |
| 1 | 2015 | Ahmed et al. (1) | article | HER2 | sarcoma | Human Epidermal Growth Factor Receptor 2 (HER2) –Specific Chimeric Antigen Receptor–Modified T Cells for the Immunotherapy of HER2-Positive Sarcoma | 20.09 |
| 2 | 2015 | Long et al. (2) | article | CD19 | hematological malignancy | 4-1BB costimulation ameliorates T cell exhaustion induced by tonic signaling of chimeric antigen receptors | 18.47 |
| 3 | 2016 | Turtle et al. (3) | article | CD19 | acute lymphoblastic leukemia | CD19 CAR–T cells of defined CD4+:CD8+ composition in adult B cell ALL patients | 11.76 |
| 4 | 2015 | Brown et al. (4) | article | IL13Rα2 | glioblastoma | Bioactivity and Safety of IL13Rα2-Redirected Chimeric Antigen Receptor CD8+ T Cells in Patients with Recurrent Glioblastoma | 11.51 |
| 5 | 2017 | O'Rourke et al. (5) | article | EGFRvIII | glioblastoma | A single dose of peripherally infused EGFRvIII-directed CAR T cells mediates antigen loss and induces adaptive resistance in patients with recurrent glioblastoma | 29.14 |
| 6 | 2016 | Brown et al. (6) | article | IL13Rα2 | glioblastoma | Regression of Glioblastoma after Chimeric Antigen Receptor T-Cell Therapy | 24.07 |
| 7 | 2017 | Ahmed et al. (7) | article | HER2 | glioblastoma | HER2-Specific Chimeric Antigen Receptor-Modified Virus-Specific T Cells for Progressive Glioblastoma: A Phase 1 Dose-Escalation Trial | 17.07 |
| 8 | 2017 | Lim et al. (8) | review |  |  | The Principles of Engineering Immune Cells to Treat Cancer | 14.15 |
| 9 | 2016 | Fesnak et al. (9) | review |  |  | Engineered T cells: the promise and challenges of cancer immunotherapy | 12.22 |
| 10 | 2018 | Maude et al. (10) | article | CD19 | acute lymphoblastic leukemia | Tisagenlecleucel in Children and Young Adults with B-Cell Lymphoblastic Leukemia | 35.58 |
| 11 | 2017 | Neelapu et al. (11) | article | CD19 | diffuse large B-cell lymphoma | Axicabtagene Ciloleucel CAR T-Cell Therapy in Refractory Large B-Cell Lymphoma | 30.82 |
| 12 | 2017 | Newick et al. (12) | review |  |  | CAR T Cell Therapy for Solid Tumors | 23.56 |
| 13 | 2018 | June et al. (13) | review |  |  | CAR T cell immunotherapy for human cancer | 21.39 |
| 14 | 2018 | June et al. (14) | review |  |  | Chimeric Antigen Receptor Therapy | 18.51 |
| 15 | 2018 | Park et al. (15) | article | CD19 | acute lymphoblastic leukemia | Long-Term Follow-up of CD19 CAR Therapy in Acute Lymphoblastic Leukemia | 18.15 |
| 16 | 2018 | Rafiq et al. (16) | article |  |  | Targeted delivery of a PD-1-blocking scFv by CAR-T cells enhances anti-tumor efficacy in vivo | 17.07 |
| 17 | 2018 | Adachi et al. (17) | article | CD20 |  | IL-7 and CCL19 expression in CAR-T cells improves immune cell infiltration and CAR-T cell survival in the tumor | 14.2 |
| 18 | 2017 | Schuster et al. (18) | article | CD19 | diffuse large B-cell lymphoma; follicular lymphoma | Chimeric Antigen Receptor T Cells in Refractory B-Cell Lymphomas | 13.94 |
| 19 | 2018 | Beatty et al. (19) | article | mesothelin | Pancreatic ductal adenocarcinoma | Activity of Mesothelin-Specific Chimeric Antigen Receptor T Cells Against Pancreatic Carcinoma Metastases in a Phase 1 Trial | 13.84 |
| 20 | 2016 | Roybal et al. (20) | article |  |  | Precision Tumor Recognition by T Cells With Combinatorial Antigen-Sensing Circuits | 12.41 |
| 21 | 2017 | Heczey et al. (21) | article | GD2 | neuroblastoma | CAR T Cells Administered in Combination with Lymphodepletion and PD-1 Inhibition to Patients with Neuroblastoma | 12.41 |
| 22 | 2018 | Fry et al. (22) | article | CD22 | acute lymphoblastic leukemia | CD22-targeted CAR T cells induce remission in B-ALL that is naive or resistant to CD19-targeted CAR immunotherapy | 11.34 |

**References:**

1. Ahmed N, Brawley VS, Hegde M, Robertson C, Ghazi A, Gerken C, et al. Human Epidermal Growth Factor Receptor 2 (HER2) -Specific Chimeric Antigen Receptor-Modified T Cells for the Immunotherapy of HER2-Positive Sarcoma. *Journal of clinical oncology : official journal of the American Society of Clinical Oncology* (2015) 33(15):1688-96. Epub 2015/03/25. doi: 10.1200/jco.2014.58.0225. PubMed PMID: 25800760; PubMed Central PMCID: PMCPMC4429176 online at <www.jco.org>. Author contributions are found at the end of this article.

2. Long AH, Haso WM, Shern JF, Wanhainen KM, Murgai M, Ingaramo M, et al. 4-1BB costimulation ameliorates T cell exhaustion induced by tonic signaling of chimeric antigen receptors. *Nature medicine* (2015) 21(6):581-90. Epub 2015/05/06. doi: 10.1038/nm.3838. PubMed PMID: 25939063; PubMed Central PMCID: PMCPMC4458184.

3. Turtle CJ, Hanafi LA, Berger C, Gooley TA, Cherian S, Hudecek M, et al. CD19 CAR-T cells of defined CD4+:CD8+ composition in adult B cell ALL patients. *The Journal of clinical investigation* (2016) 126(6):2123-38. Epub 2016/04/26. doi: 10.1172/jci85309. PubMed PMID: 27111235; PubMed Central PMCID: PMCPMC4887159.

4. Brown CE, Badie B, Barish ME, Weng L, Ostberg JR, Chang WC, et al. Bioactivity and Safety of IL13Rα2-Redirected Chimeric Antigen Receptor CD8+ T Cells in Patients with Recurrent Glioblastoma. *Clinical cancer research : an official journal of the American Association for Cancer Research* (2015) 21(18):4062-72. Epub 2015/06/11. doi: 10.1158/1078-0432.Ccr-15-0428. PubMed PMID: 26059190; PubMed Central PMCID: PMCPMC4632968.

5. O'Rourke DM, Nasrallah MP, Desai A, Melenhorst JJ, Mansfield K, Morrissette JJD, et al. A single dose of peripherally infused EGFRvIII-directed CAR T cells mediates antigen loss and induces adaptive resistance in patients with recurrent glioblastoma. *Science translational medicine* (2017) 9(399). Epub 2017/07/21. doi: 10.1126/scitranslmed.aaa0984. PubMed PMID: 28724573; PubMed Central PMCID: PMCPMC5762203.

6. Brown CE, Alizadeh D, Starr R, Weng L, Wagner JR, Naranjo A, et al. Regression of Glioblastoma after Chimeric Antigen Receptor T-Cell Therapy. *The New England journal of medicine* (2016) 375(26):2561-9. Epub 2016/12/29. doi: 10.1056/NEJMoa1610497. PubMed PMID: 28029927; PubMed Central PMCID: PMCPMC5390684.

7. Ahmed N, Brawley V, Hegde M, Bielamowicz K, Kalra M, Landi D, et al. HER2-Specific Chimeric Antigen Receptor-Modified Virus-Specific T Cells for Progressive Glioblastoma: A Phase 1 Dose-Escalation Trial. *JAMA oncology* (2017) 3(8):1094-101. Epub 2017/04/21. doi: 10.1001/jamaoncol.2017.0184. PubMed PMID: 28426845; PubMed Central PMCID: PMCPMC5747970.

8. Lim WA, June CH. The Principles of Engineering Immune Cells to Treat Cancer. *Cell* (2017) 168(4):724-40. Epub 2017/02/12. doi: 10.1016/j.cell.2017.01.016. PubMed PMID: 28187291; PubMed Central PMCID: PMCPMC5553442.

9. Fesnak AD, June CH, Levine BL. Engineered T cells: the promise and challenges of cancer immunotherapy. *Nature reviews Cancer* (2016) 16(9):566-81. Epub 2016/08/24. doi: 10.1038/nrc.2016.97. PubMed PMID: 27550819; PubMed Central PMCID: PMCPMC5543811.

10. Maude SL, Laetsch TW, Buechner J, Rives S, Boyer M, Bittencourt H, et al. Tisagenlecleucel in Children and Young Adults with B-Cell Lymphoblastic Leukemia. *The New England journal of medicine* (2018) 378(5):439-48. Epub 2018/02/01. doi: 10.1056/NEJMoa1709866. PubMed PMID: 29385370; PubMed Central PMCID: PMCPMC5996391.

11. Neelapu SS, Locke FL, Bartlett NL, Lekakis LJ, Miklos DB, Jacobson CA, et al. Axicabtagene Ciloleucel CAR T-Cell Therapy in Refractory Large B-Cell Lymphoma. *The New England journal of medicine* (2017) 377(26):2531-44. Epub 2017/12/12. doi: 10.1056/NEJMoa1707447. PubMed PMID: 29226797; PubMed Central PMCID: PMCPMC5882485.

12. Newick K, O'Brien S, Moon E, Albelda SM. CAR T Cell Therapy for Solid Tumors. *Annual review of medicine* (2017) 68:139-52. Epub 2016/11/20. doi: 10.1146/annurev-med-062315-120245. PubMed PMID: 27860544.

13. June CH, O'Connor RS, Kawalekar OU, Ghassemi S, Milone MC. CAR T cell immunotherapy for human cancer. *Science (New York, NY)* (2018) 359(6382):1361-5. Epub 2018/03/24. doi: 10.1126/science.aar6711. PubMed PMID: 29567707.

14. June CH, Sadelain M. Chimeric Antigen Receptor Therapy. *The New England journal of medicine* (2018) 379(1):64-73. Epub 2018/07/05. doi: 10.1056/NEJMra1706169. PubMed PMID: 29972754; PubMed Central PMCID: PMCPMC7433347.

15. Park JH, Rivière I, Gonen M, Wang X, Sénéchal B, Curran KJ, et al. Long-Term Follow-up of CD19 CAR Therapy in Acute Lymphoblastic Leukemia. *The New England journal of medicine* (2018) 378(5):449-59. Epub 2018/02/01. doi: 10.1056/NEJMoa1709919. PubMed PMID: 29385376; PubMed Central PMCID: PMCPMC6637939.

16. Rafiq S, Yeku OO, Jackson HJ, Purdon TJ, van Leeuwen DG, Drakes DJ, et al. Targeted delivery of a PD-1-blocking scFv by CAR-T cells enhances anti-tumor efficacy in vivo. *Nature biotechnology* (2018) 36(9):847-56. Epub 2018/08/14. doi: 10.1038/nbt.4195. PubMed PMID: 30102295; PubMed Central PMCID: PMCPMC6126939.

17. Adachi K, Kano Y, Nagai T, Okuyama N, Sakoda Y, Tamada K. IL-7 and CCL19 expression in CAR-T cells improves immune cell infiltration and CAR-T cell survival in the tumor. *Nature biotechnology* (2018) 36(4):346-51. Epub 2018/03/06. doi: 10.1038/nbt.4086. PubMed PMID: 29505028.

18. Schuster SJ, Svoboda J, Chong EA, Nasta SD, Mato AR, Anak Ö, et al. Chimeric Antigen Receptor T Cells in Refractory B-Cell Lymphomas. *The New England journal of medicine* (2017) 377(26):2545-54. Epub 2017/12/12. doi: 10.1056/NEJMoa1708566. PubMed PMID: 29226764; PubMed Central PMCID: PMCPMC5788566.

19. Beatty GL, O'Hara MH, Lacey SF, Torigian DA, Nazimuddin F, Chen F, et al. Activity of Mesothelin-Specific Chimeric Antigen Receptor T Cells Against Pancreatic Carcinoma Metastases in a Phase 1 Trial. *Gastroenterology* (2018) 155(1):29-32. Epub 2018/03/24. doi: 10.1053/j.gastro.2018.03.029. PubMed PMID: 29567081; PubMed Central PMCID: PMCPMC6035088.

20. Roybal KT, Rupp LJ, Morsut L, Walker WJ, McNally KA, Park JS, et al. Precision Tumor Recognition by T Cells With Combinatorial Antigen-Sensing Circuits. *Cell* (2016) 164(4):770-9. Epub 2016/02/03. doi: 10.1016/j.cell.2016.01.011. PubMed PMID: 26830879; PubMed Central PMCID: PMCPMC4752902.

21. Heczey A, Louis CU, Savoldo B, Dakhova O, Durett A, Grilley B, et al. CAR T Cells Administered in Combination with Lymphodepletion and PD-1 Inhibition to Patients with Neuroblastoma. *Molecular therapy : the journal of the American Society of Gene Therapy* (2017) 25(9):2214-24. Epub 2017/06/13. doi: 10.1016/j.ymthe.2017.05.012. PubMed PMID: 28602436; PubMed Central PMCID: PMCPMC5589058.

22. Fry TJ, Shah NN, Orentas RJ, Stetler-Stevenson M, Yuan CM, Ramakrishna S, et al. CD22-targeted CAR T cells induce remission in B-ALL that is naive or resistant to CD19-targeted CAR immunotherapy. *Nature medicine* (2018) 24(1):20-8. Epub 2017/11/21. doi: 10.1038/nm.4441. PubMed PMID: 29155426; PubMed Central PMCID: PMCPMC5774642.
